# Combinatorial transcriptomic and genetic dissection of insulin/IGF‐1 signaling‐regulated longevity in *Caenorhabditis elegans*

**DOI:** 10.1111/acel.14151

**Published:** 2024-03-26

**Authors:** Seokjin Ham, Sieun S. Kim, Sangsoon Park, Hyunwoo C. Kwon, Seokjun G. Ha, Yunkyu Bae, Gee‐Yoon Lee, Seung‐Jae V. Lee

**Affiliations:** ^1^ Department of Biological Sciences Korea Advanced Institute of Science and Technology Daejeon South Korea

**Keywords:** *Caenorhabditis elegans*, *daf‐2*, insulin/IGF‐1 signaling, longevity, transcriptome

## Abstract

Classical genetic analysis is invaluable for understanding the genetic interactions underlying specific phenotypes, but requires laborious and subjective experiments to characterize polygenic and quantitative traits. Contrarily, transcriptomic analysis enables the simultaneous and objective identification of multiple genes whose expression changes are associated with specific phenotypes. Here, we conducted transcriptomic analysis of genes crucial for longevity using datasets with *daf‐2*/insulin/IGF‐1 receptor mutant *Caenorhabditis elegans*. Our analysis unraveled multiple epistatic relationships at the transcriptomic level, in addition to verifying genetically established interactions. Our combinatorial analysis also revealed transcriptomic changes associated with longevity conferred by *daf‐2* mutations. In particular, we demonstrated that the extent of lifespan changes caused by various mutant alleles of the longevity transcription factor *daf‐16*/*FOXO* matched their effects on transcriptomic changes in *daf‐2* mutants. We identified specific aging‐regulating signaling pathways and subsets of structural and functional RNA elements altered by different genes in *daf‐2* mutants. Lastly, we elucidated the functional cooperation between several longevity regulators, based on the combination of transcriptomic and molecular genetic analysis. These data suggest that different biological processes coordinately exert their effects on longevity in biological networks. Together our work demonstrates the utility of transcriptomic dissection analysis for identifying important genetic interactions for physiological processes, including aging and longevity.

## Graphical abstract

Here we present combinatorial transcriptomic and genetic dissection of longevity regulators crucial for reduced insulin/IGF‐1 signaling (IIS)‐mediated longevity in *C. elegans*. Our work demonstrates the utility of transcriptomic dissection analysis for identifying important genetic interactions for physiological processes, including aging and longevity.

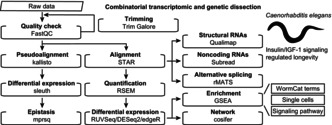

**Abbreviations**
cosifer: COnSensus Interaction Network InFErence Service
DDX39A: DExD‐box helicase 39A
DEG: differentially expressed gene
DESeq2: Differential Expression analysis for Sequence count data 2
edgeR: Empirical Analysis of Digital Gene Expression Data in R
ENCODE: Encyclopedia of DNA Elements
FOXO: forkhead box O
GSEA: gene set enrichment analysis
HSF1: heat shock transcription factor 1
IGF‐1: insulin‐like growth factor 1
IGV: Integrative Genomics Viewer
IIS: insulin/IGF‐1signaling
lincRNA: long intergenic noncoding RNA
MDS: multiple dimensional scaling
mprsq: Massively Parallel RNA‐seq Project
NES: normalized enrichment scores
NGM: nematodegrowth media
NRF: nuclear respiratory factor
OASIS2: online application of the survival analysis 2
PFDN6: prefoldin subunit 6
PIP3: phosphatidylinositol (3,4,5)‐trisphosphate
PTEN: phosphatase and tensin homolog
REST: RE1 silencing transcription factor
rlog: regularized log
rMATS: replicate Multivariate Analysis of Transcript Splicing
RNA‐seq: RNA sequencing
RSEM: RNA‐Seq by Expectation‐Maximization
RUVg: Remove Unwanted Variation Using Control Genes
RUVSeq: Remove Unwanted Variation from RNA‐Seq Data
SMARCC: SWI/SNF‐related matrix associated actin‐dependent regulator of chromatinsubfamily c member
STAR: Spliced Transcripts Alignment to a Reference
TFEB: transcription factor EB
USP7: ubiquitin specific peptidase 7
WormCat: WormCatalog
WT: wild‐type

## 1 INTRODUCTION

Epistasis is defined as the interaction of genetic changes in two or more genes, which are determined by analyzing phenotypes (Bateson et al., 1909; Phillips, 2008). Epistasis includes hierarchical relationships and non‐hierarchical relationships, such as feedback and feedforward loops (Azpeitia et al., 2011). Classical epistasis analysis is useful for understanding the genetic basis of the signaling and molecular interactions underlying particular phenotypes, but it has several limitations. For example, phenotypic epistasis analysis usually requires extensive and subjective experiments and a large number of individuals to detect significant genetic interactions. Analysis of polygenic and quantitative traits also presents challenges in identifying the number of genes that participate in specific interactions. In addition, classical epistasis analysis has difficulty analyzing non‐binary phenotypes, including partial suppression (Avery & Wasserman, 1992). Therefore, effective strategies are required to overcome the limitations of phenotype‐based classical epistasis analysis.

Transcriptomic analysis using microarray and/or RNA sequencing (RNA‐seq) (Heller, 2002; Wang et al., 2009; Wilhelm & Landry, 2009) has been applied to systematically characterize the interactions of genes crucial for various biological processes such as neuronal activities, hypoxia‐inducible factor 1‐regulated responses to low oxygen levels, pathogenic avoidance, aging, and immunosenescence (Angeles‐Albores et al., 2018; Ham et al., 2022; Kaletsky et al., 2016, 2020; Lee et al., 2021). Transcriptomic dissection of phenotypes offers several advantages over classical genetic epistasis analysis. For example, transcriptomic analysis is adequate for the identification of many genes whose expression changes influence specific phenotypes (Garber et al., 2011; Wang et al., 2009; Wilhelm & Landry, 2009). Transcriptomic dissection analysis also provides quantitative measurements of genetic interactions in complex phenotypes (Angeles‐Albores et al., 2018; Evans et al., 2023). Thus, transcriptomic analysis of phenotypes represents an effective strategy for identifying multiple previously undetected genetic interactions.

*Caenorhabditis elegans* is an excellent animal model for the genetic analyses of various physiological processes, including development, behavior, metabolism, and aging. After the DAF‐2/insulin/insulin‐like growth factor 1 (IGF‐1) receptor was discovered as a key mediator of longevity in *C. elegans* (Kenyon et al., 1993), its downstream components in the insulin/IGF‐1 signaling (IIS) pathway have been extensively studied using various strategies, including genetic epistasis analysis (Altintas et al., 2016; Lee & Lee, 2022; Murphy & Hu, 2013). For example, mutations in *daf‐18*/phosphatase and tensin homolog (*PTEN*) and *daf‐16*/forkhead box O (*FOXO*), which act downstream of the DAF‐2/insulin/IGF‐1 receptor, abolish the increased lifespan caused by mutations in *daf‐2* (Gil et al., 1999; Lin et al., 1997, 2001; Mihaylova et al., 1999; Ogg et al., 1997; Ogg & Ruvkun, 1998); DAF‐18/PTEN dephosphorylates phosphatidylinositol (3,4,5)‐trisphosphate (PIP_3_) to consequently antagonize the deactivation of DAF‐16/FOXO (Gil et al., 1999; Maehama & Dixon, 1998; Mihaylova et al., 1999; Ogg & Ruvkun, 1998). The role of these key factors in longevity has been established at the molecular level, including at the isoform level for DAF‐16/FOXO. For example, specific genetic inhibition of the *daf‐16a* isoform substantially decreases lifespan extension caused by *daf‐2* mutations, whereas that of the *daf‐16f* isoform has a small effect on longevity (Chen et al., 2015). Contrarily, the contribution of *daf‐16b* to longevity is marginal (Kwon et al., 2010; Lee et al., 2001; Lin et al., 2001). Compared with extensive classical genetic analysis, transcriptomic analysis of aging and lifespan phenotypes in *C. elegans* remains largely unexplored.

In this study, we aimed at comprehensive dissection of genetic interactions within the IIS pathway by analyzing the transcriptome of double and triple mutants in the *daf‐2* mutant background. We verified established genetic interactions and sought to unveil unexpected epistatic relationships among downstream components in the IIS pathway. We revealed that the extent of lifespan changes caused by various *daf‐16* mutant alleles correlated with those of transcriptomic changes in *daf‐2* mutants. By analyzing RNA elements affected by different genetic interventions, we found that several genetic alterations affected specific subsets of structural and functional RNA elements in *daf‐2* mutants. Finally, we identified and experimentally validated several unexpected genetic interactions between longevity regulators, which act coordinately to contribute to longevity in *daf‐2* mutants. Overall, our study demonstrates that transcriptomic dissection analysis is useful for identifying genetic interactions for physiological processes and complements phenotype‐based classical genetic approaches.

## 2 METHODS

### 2.1 Strains

All strains were maintained under standard laboratory culture conditions on nematode growth media (NGM) seeded with *E. coli* OP50. Strains that were used in this study were outcrossed at least four times to wild‐type (WT) N2 strain. The list of strains used in this study is described as follows: WT N2, CF1041 *daf‐2* *(* *e1370* *)* *III*, IJ445 *smg‐2* *(* *qd101* *)* *I* obtained by outcrossing ZD627 four times to Lee‐laboratory N2, IJ446 *smg‐2* *(* *qd101* *)* *I; daf‐2* *(* *e1370* *)* *III* obtained by crossing IJ445 and CF1041, IJ1906 *hlh‐30* *(* *tm1978* *)* *IV* obtained by outcrossing JIN1375 six times to Lee‐laboratory N2, IJ2078 *daf‐2* *(* *e1370* *)* *III; hlh‐30* *(* *tm1978* *)* *IV* obtained by crossing IJ1906 and CF1041, IJ438 *smg‐2* *(* *qd101* *)* *I; daf‐2* *(* *e1370* *)* *III; hlh‐30* *(* *tm1978* *)* *IV* obtained by crossing IJ446 and IJ1906, IJ1859 *sqIs17* *[* *hlh‐30p::hlh‐30::GFP; rol‐6* *(* *su1006* *)* *]* obtained by outcrossing MAH240 four times to Lee‐laboratory N2, IJ71 *smg‐2* *(* *qd101* *)* *I; sqIs17* *[* *hlh‐30p::hlh‐30::GFP; rol‐6* *(* *su1006* *)* *]* obtained by crossing IJ1859 and IJ445, IJ1228 *yhEx330* *[* *smg‐1p::smg‐1::GFP; odr‐1p::RFP* *]*, IJ70 *hlh‐30* *(* *tm1978* *)* *IV; yhEx330* *[* *smg‐1p::smg‐1::GFP; odr‐1p::RFP* *]* obtained by crossing IJ1228 and IJ1906.

### 2.2 Preprocessing of RNA‐seq data for transcriptomic analysis

Alignment and quantification of RNA‐seq data were performed by adopting parameters described in the guidelines of the Encyclopedia of DNA Elements (ENCODE) long RNA‐Seq processing pipeline (https://www.encodeproject.org/pipelines/ENCPL002LPE/). Sequencing reads were aligned to the *C. elegans* genome WBcel235 (ce11) and Ensembl transcriptome (release 103) by using the Spliced Transcripts Alignment to a Reference (STAR) (v.2.7.0e) (Dobin et al., 2013). After the alignment, genotypes of datasets were confirmed by using the Integrative Genomics Viewer (IGV) (v.2.14.1) (Thorvaldsdottir et al., 2013). Gene expression levels were quantified with the aligned reads by using the RNA‐Seq by Expectation–Maximization (RSEM) (v.1.3.1) (Li & Dewey, 2011). The raw counts were used for the identification of differentially expressed genes (DEGs). Genes with fold change >2 and adjusted *p* < 0.05 were identified as DEGs by using Differential Expression analysis for Sequence count data 2 (DESeq2) (v.1.28.1) (Love et al., 2014) unless otherwise noted. For datasets without replicates, DEGs with fold change > 2 and adjusted *p* < 0.05 were detected by using empirically estimated biological coefficient of variation of 0.1 and the Empirical Analysis of Digital Gene Expression Data in R (edgeR) (v.3.30.3) (McCarthy et al., 2012; Robinson et al., 2010). Wald test *p* values were adjusted for multiple testing using the procedure of Benjamini and Hochberg (Benjamini & Hochberg, 1995).

### 2.3 Transcriptomic epistasis

The epistasis analysis of RNA‐seq data is composed of WT animals (control), single mutants (A and B), and double mutants (AB). Transcript levels were quantified with *C. elegans* Ensembl transcriptome (release 103) by using kallisto (v.0.46.0) (Bray et al., 2016). Differential levels of transcript isoforms were analyzed by using the likelihood ratio test of sleuth (v.0.30.0) (Pimentel et al., 2017). Single coefficients to quantify transcriptome‐wide epistasis of transcript isoforms with adjusted *p* < 0.1 were calculated by using the Massively Parallel RNA‐seq Project (mprsq) (Angeles‐Albores et al., 2018) based on the guidelines (https://wormlabcaltech.github.io/mprsq/analysis_notebooks/6_epistasis.html). *p* values were adjusted for multiple testing using the procedure of Benjamini and Hochberg (Benjamini & Hochberg, 1995).

### 2.4 Global comparisons of gene expression levels

Confounding factors between different datasets were adjusted by upper quartile normalization followed by the Remove Unwanted Variation from RNA‐Seq Data (RUVSeq) (v.1.22.0) (Risso et al., 2014). In particular, the Remove Unwanted Variation Using Control Genes (RUVg) was used and genes with nominal *p* > 0.1 were defined as in silico empirical negative controls. After the removal, the counts were converted to regularized log (rlog) values (Love et al., 2014) for multiple dimensional scaling using two dimensions with relative Euclidian distances and a pairwise comparison matrix using Pearson's correlation coefficients. Overlapping targets of longevity regulators were compared by using UpSet plots (Lex et al., 2014).

### 2.5 Gene set enrichment analysis

Biological terms enriched in genes of interest were identified by using the WormCatalog (WormCat) (Higgins et al., 2022; Holdorf et al., 2020). Global expression changes of the WormCat terms and target genes of certain transcription factors caused by the genetic inhibition of different components were represented as normalized enrichment scores (NES) by using GSEA (v.3.0) (Subramanian et al., 2005). Biomarkers of individual cell types defined by single cell RNA‐seq data analysis of wild‐type and *daf‐2* mutants (Preston et al., 2019) were used for the single cell level transcriptome analysis. Cumulative fractions with expression levels of genes of interest were also used for the global comparisons. R (v.4.1.0, http://www.r‐project.org) was used for all the data plotting and statistical tests unless stated otherwise.

### 2.6 Analysis of changes in specific functional and structural RNAs

The reads aligned to annotated exons, introns, and intergenic regions were analyzed by using Qualimap (v.2.2.1) (Okonechnikov et al., 2016). Long intergenic noncoding RNAs (Akay et al., 2019) were quantified by using featureCounts command in Subread package (v.2.0.0) (Liao et al., 2014). Changes in alternative splicing events were analyzed by using the replicate Multivariate Analysis of Transcript Splicing (rMATS) (v.4.1.2) (Shen et al., 2014), and the changes with nominal *p* < 0.05 were defined as significant ones. Reads that span junctions and reads within exons were counted.

### 2.7 Reconstruction of networks using a consensus approach

From log_2_ transformed fold changes of genes compared to controls, intensities of links between different genetic components were calculated by using the COnSensus Interaction Network InFErence Service (cosifer) (Manica et al., 2021) with following parameters: –no‐standardize –samples_on_rows –index 0 –combiner summa. The networks were visualized by using Cytoscape (v. 3.10.0) (Shannon et al., 2003) with yFiles organic layout.

### 2.8 Lifespan assays

Lifespan assays were performed at 20°C on NGM plates seeded with OP50 for experiments with mutants as described previously with minor modifications (Jung et al., 2021). RNAi‐mediated lifespan assays were performed at 20°C on NGM plates containing 1 mM of isopropyl β‐D‐1‐thiogalactopyranoside (IPTG; Gold Biotechnology, St. Louis, MO, USA) and 100 μg/mL ampicillin (Thermo Fisher Scientific, Waltham, MA, USA) seeded with HT115 bacteria expressing double‐stranded RNA targeting *pfd‐6*. HT115 bacteria were cultured in Luria broth (LB) containing 100 μg/mL ampicillin overnight at 37°C. One hundred μL of bacteria was seeded on plates and incubated overnight at 37°C. One mM of IPTG was added and incubated at room temperature for 24 hours before use (Ham et al., 2022; Park et al., 2022). Briefly, synchronized prefertile young adult animals were transferred to plates containing 5 μM 5‐fluoro‐2'‐deoxyuridine (FUDR; Sigma‐Aldrich, MO, USA) to prevent progeny from hatching. All lifespan assays were conducted by at least two independent researchers, and a minimum of four plates were used for each condition, except for Figure 6d,e. Animals that ruptured, displayed internal hatching, or crawled off the plates were censored but included in the lifespan analysis as censored animals. Statistical analysis of the lifespan data was conducted using online application of the survival analysis 2 (OASIS2, http://sbi.postech.ac.kr/oasis2) (Han et al., 2016). *p* values were calculated using the log‐rank (Mantel–Cox) test.

## 3 RESULTS

### 3.1 Transcriptomic epistasis analysis between *daf‐2* and various genes whose depletion suppresses longevity

**FIGURE 1:**
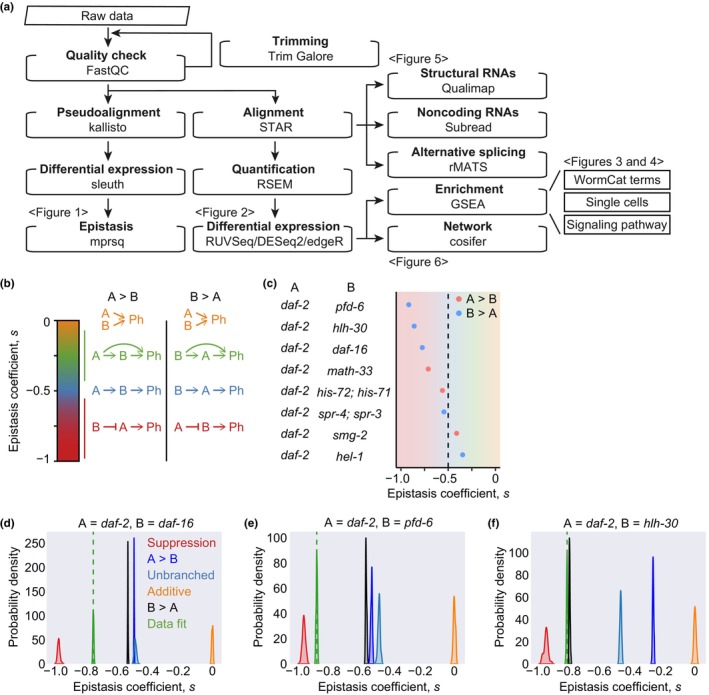
Transcriptomic epistasis analysis between *daf‐2* and various genes crucial for reduced insulin/IGF‐1 signaling (IIS)‐mediated longevity. (a) Overall workflow of combinatorial transcriptomic and genetic dissection analyses. See methods for particular analysis tools that were used in this study. (b) Schematic diagram of transcriptomic epistasis between A and B genotypes for a certain phenotype (Ph) (Angeles‐Albores et al., 2018). Epistasis coefficient, (*s*) = 0: additive (orange), −1/2 < *s* < 0: branched (green), *s* = −1/2: unbranched (blue), *s* = −1: repressive (red). A > B indicates that the phenotype of a single mutant A is similar to that of the double mutant AB, whereas B > A suggests the opposite. (c) Epistasis coefficients between *daf‐2* and various genes whose depletion suppresses the longevity of *daf‐2* mutants. (d–f) Comparison of simulated epistasis coefficients against the observed coefficients between *daf‐2* and *daf‐16* (d), *pfd‐6* (e), and *hlh‐30* (f). Distribution was calculated based on empirical bootstrapping. A dashed green line indicates the average of the data. Datasets include RNA‐seq data using *daf‐2* mutants in combination with genetic inhibition of PFD‐6/PFDN6 (Son et al., 2018), HLH‐30/TFEB (Lin et al., 2018), DAF‐16/FOXO (Lin et al., 2018), MATH‐33/USP7 (Heimbucher et al., 2015), HIS‐71 and HIS‐72/H3.3 (Piazzesi et al., 2016), SPR‐3 and SPR‐4/REST (Zullo et al., 2019) SMG‐2/UPF1 (Son et al., 2017), and HEL‐1/DDX39A (Seo et al., 2015).

We aimed at comprehensive understanding of genetic interactions within the IIS pathway by using combinatorial transcriptomic and genetic dissection analyses (Figure 1a). We performed transcriptomic epistasis analysis, as described previously (Angeles‐Albores et al., 2018), between *daf‐2* and genetic factors whose depletion decreases longevity caused by *daf‐2* mutations. Specifically, we calculated the epistasis coefficient between *daf‐2* (A) and different genes (B), *s* (A, B), and sought to dissect their relationships (Figure 1b–f; Figure S1). The analysis required RNA‐seq data from wild‐type animals (control), single mutants (A and B), and double mutants (AB) with phenotype (Ph) as an outcome. These include genetic inhibition of PFD‐6/prefoldin subunit 6 (PFDN6) (Son et al., 2018), HLH‐30/transcription factor EB (TFEB) (Lin et al., 2018), DAF‐16/FOXO (Lin et al., 2018), MATH‐33/ubiquitin specific peptidase 7 (USP7) (Heimbucher et al., 2015), HIS‐71/histone variant H3.3 and HIS‐72/histone variant H3.3 (Piazzesi et al., 2016), SPR‐3/RE1 silencing transcription factor (REST) and SPR‐4/REST (Zullo et al., 2019), SMG‐2/Up‐frameshift protein 1 (UPF1) RNA helicase (Son et al., 2017), and HEL‐1/DExD‐box helicase 39A (DDX39A) (Seo et al., 2015) (Figure 1c; Appendix S1). We obtained an epistasis coefficient of −0.77 between *daf‐2* and *daf‐16* and found that *daf‐16* mutant phenotypes were similar to those of *daf‐16; daf‐2* double mutants (*daf‐16* > *daf‐2*) (Figure 1c,d). This is consistent with the established findings that DAF‐2 inhibits DAF‐16 (Altintas et al., 2016; Gems et al., 1998; Gottlieb & Ruvkun, 1994; Kenyon et al., 1993; Lee & Lee, 2022; Murphy et al., 2003; Vowels & Thomas, 1992). We also obtained similar results for *pfd‐6* and *hlh‐30* (Figure 1c,e,f), consistent with previous reports illustrating that DAF‐2 inhibits PFD‐6 and HLH‐30 (Lin et al., 2018; Son et al., 2018). Overall, our transcriptomic epistasis analysis verified established genetic epistasis.

Our analysis also highlighted unexpected possibilities for epistatic relationships. Our analysis raises the possibility that MATH‐33 inhibits DAF‐2 (Figure 1c; Figure S1c). In addition, HIS‐71/HIS‐72, SPR‐3/SPR‐4, and SMG‐2 appear to act with DAF‐2 in a linear pathway (Figure 1c; Figure S1d–f). Moreover, HEL‐1 might upregulate DAF‐2 via a feedforward pathway (Figure 1c; Figure S1g). Collectively, these results suggest that transcriptomic epistasis analysis is useful for confirming established genetic interactions and suggesting unexpected relationships among genes.

### 3.2 Transcriptomic dissection analysis among factors that contribute to longevity associated with *daf‐2* mutations

**FIGURE 2:**
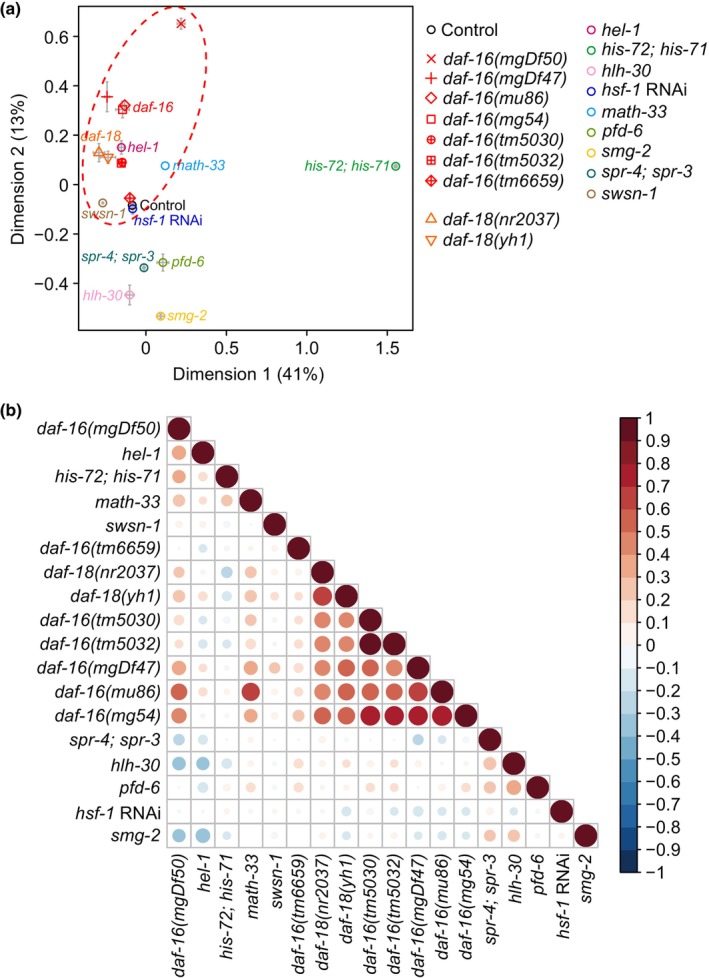
Transcriptomic dissection of factors that mediate longevity caused by *daf‐2* mutations. (a) A multidimensional scaling plot showing relative Euclidian distances of top 500 genes among various genetic factors that contribute to the longevity of *daf‐2* mutants (Control) at transcriptome levels after regularized log transformation (Love et al., 2014) and batch effect correction (Risso et al., 2014). A dotted red oval represents datasets of multiple *daf‐16*, *daf‐18*, *hel‐1*, *swsn‐1*, and *math‐33* mutant alleles that clustered together. (b) Pearson's correlation coefficients of transcriptome levels using colors (red: positive, blue: negative) changed by the analyzed genetic factors in *daf‐2* mutant backgrounds. Datasets include RNA‐seq data with *daf‐2* mutants in combination with genetic inhibition of DAF‐16/FOXO (Chen et al., 2015; Heimbucher et al., 2015; Kumar et al., 2015; Lin et al., 2018; Riedel et al., 2013), DAF‐18/PTEN (Park et al., 2021), HEL‐1/DDX39A (Seo et al., 2015), HIS‐71 and HIS‐72/H3.3 (Piazzesi et al., 2016), HLH‐30/TFEB (Lin et al., 2018), HSF‐1/HSF1 (Lee et al., 2021), MATH‐33/USP7 (Heimbucher et al., 2015), PFD‐6/PFDN6 (Son et al., 2018), SMG‐2/UPF1 (Son et al., 2017), SPR‐3 and SPR‐4/REST (Zullo et al., 2019), and SWSN‐1/SMARCC (Riedel et al., 2013). See Appendix S3 for detailed values used for this figure.

Despite its usefulness, analysis using epistasis coefficients is challenging for identifying relationships among three or more components in a pathway because of the combinatorial explosion of interactions (Moore & Williams, 2009). In addition, epistasis coefficients cannot be obtained if any of the data of single and double mutants are missing. We therefore performed combinatorial analysis of RNA‐seq datasets using genetic inhibition of 11 genes, which decreases longevity caused by *daf‐2* mutations. These include genetic inhibition of DAF‐16/FOXO (Chen et al., 2015; Heimbucher et al., 2015; Kumar et al., 2015; Lin et al., 2018; Riedel et al., 2013), DAF‐18/PTEN (Park et al., 2021), HEL‐1/DDX39A (Seo et al., 2015), HIS‐71/H3.3 and HIS‐72/H3.3 (Piazzesi et al., 2016), HLH‐30/TFEB (Lin et al., 2018), HSF‐1/heat shock transcription factor 1 (HSF1) (Lee et al., 2021), MATH‐33/USP7 (Heimbucher et al., 2015), PFD‐6/PFDN6 (Son et al., 2018), SMG‐2/UPF1 (Son et al., 2017), SPR‐3/REST and SPR‐4/REST (Zullo et al., 2019), and SWSN‐1/SWI/SNF‐related matrix associated actin‐dependent regulator of chromatin subfamily c member (SMARCC) (Riedel et al., 2013), in the *daf‐2* mutant background (Appendices S1 and S2). We displayed datasets on multiple dimensional scaling (MDS) using two dimensions with relative Euclidian distances (Figure 2a) and a pairwise comparison matrix using Pearson's correlation coefficients (Figure 2b). As expected, we found that the datasets of multiple *daf‐16* mutant alleles clustered together (Figure 2; Appendix S3). In addition, we showed that mutations in *daf‐18*, which acts upstream of DAF‐16 and downstream of DAF‐2 (Gil et al., 1999; Mihaylova et al., 1999; Ogg & Ruvkun, 1998), affected the transcriptome at moderate levels along the trajectory formed by the *daf‐16* mutations in the MDS (Figure 2a). In addition, the datasets of *daf‐18* mutations clustered with those of *daf‐16* mutations in the pairwise comparison matrix (Figure 2b). HEL‐1, an RNA helicase, and SWSN‐1, a component of the chromatin remodeler SWI/SNF complex, are recruited by and necessary for DAF‐16 target gene activation (Riedel et al., 2013; Seo et al., 2015). MATH‐33 is responsible for stabilizing DAF‐16 (Heimbucher et al., 2015). Consistent with these reports, mutations in *hel‐1*, *swsn‐1*, and *math‐33* elicited transcriptomic changes similar to those caused by *daf‐16* mutations compared to mutations of other genes, including *hlh‐30*, *smg‐2*, and *pfd‐6* (Figure 2; Appendix S3). We also found that genes that were upregulated by DAF‐18 and MATH‐33 were similar to those by DAF‐16 in *daf‐2* mutants (Figure S2; Appendix S4). Contrarily, mutations in *hlh‐30*, *spr‐3*/*spr‐4*, *pfd‐6*, and *smg‐2* elicited transcriptomic changes distinct from those caused by *daf‐16* mutations (Figure 2). Overall, these data indicate that the effects of mutations in genes encoding established longevity proteins, including DAF‐18, HEL‐1, SWSN‐1, and MATH‐33, that work with DAF‐16 on the transcriptome of *daf‐2* mutants are similar to those of *daf‐16* mutations.

### 3.3 The extent of lifespan changes caused by various *daf‐16* mutant alleles correlates with their transcriptomic changes in *daf‐2* mutants

**FIGURE 3:**
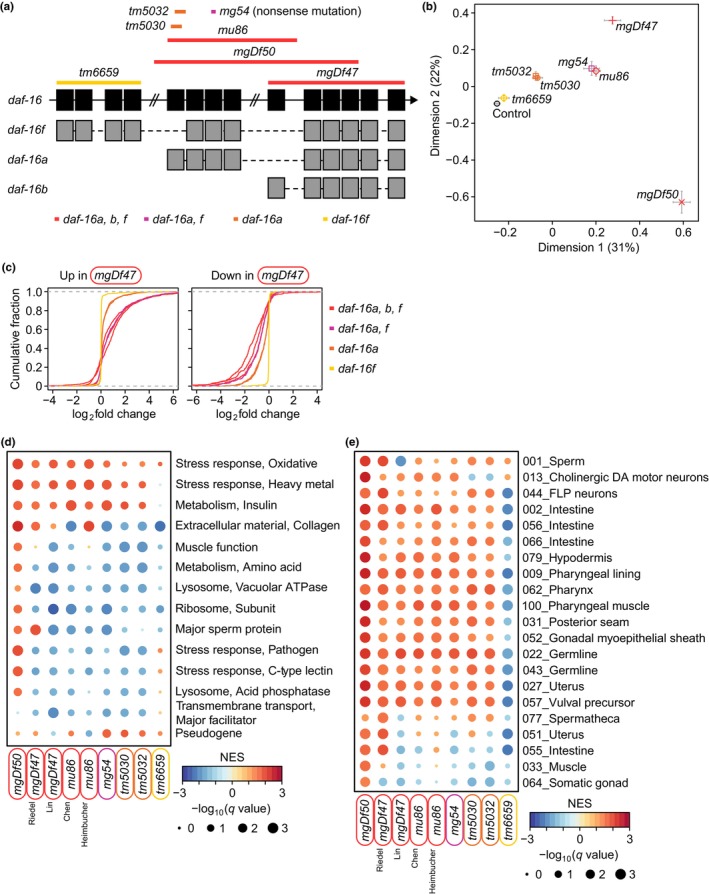
Transcriptomic analysis of seven selected *daf‐16* mutant alleles in *daf‐2* mutant backgrounds. (a) Schematic diagram of the *daf‐16* mutant alleles along *daf‐16* gene (black) and *daf‐16* transcript isoforms (gray). Colors of the alleles indicate the extents of their impacts on phenotypic and transcriptomic changes: red > pink > orange > yellow. (b) A multidimensional scaling plot showing relative Euclidian distances among the seven *daf‐16* mutant alleles that we analyzed in *daf‐2* mutant backgrounds at transcriptome levels. (c) Cumulative fraction of the genes in an ascending order of the extent of gene expression changes conferred by the *daf‐16* mutant alleles in *daf‐2* mutant backgrounds. *daf‐16* isoforms that are affected by mutant alleles are indicated. (d) Normalized enrichment scores (NES) of transcriptomic changes in indicated WormCat terms at intermediate levels caused by various *daf‐16* mutant alleles. *q*‐values were obtained by calculating the false discovery rate corresponding to each NES. Representative terms were selected based on minimum *q*‐values (*q* = 0.05) among different conditions. The terms were further selected if at least four conditions showed absolute NES greater than 1.5. Terms were sorted based on the number of upregulated terms. (e) NES of transcriptomic changes for biomarkers elicited by various *daf‐16* mutant alleles in indicated cells (Preston et al., 2019). NES range from −3 (blue) to +3 (red). The size of black circles correlates with −log_10_(*q*‐value). References for datasets with the same mutant alleles were indicated as follows: ^Riedel^ *mgDf47* (Riedel et al., 2013), ^Lin^ *mgDf47* (Lin et al., 2018), ^Chen^ *mu86* (Chen et al., 2015), and ^Heimbucher^ *mu86* (Heimbucher et al., 2015).

In *C. elegans*, *daf‐16* is expressed as multiple isoforms to regulate various IIS‐mediated processes, including longevity (Chen et al., 2015; Kwon et al., 2010). To examine whether the roles of distinct isoforms elicited different transcriptomic changes, we analyzed the allelic nature of seven different *daf‐16* mutations, *mgDf50*, *mgDf47*, *mu86*, *mg54*, *tm5030*, *tm5032*, and *tm6659*, at the transcriptomic level (Figure 2a). Three functionally null or strong loss‐of‐function alleles, *mgDf50*, *mgDf47*, and *mu86*, delete parts of the *a*, *b*, and *f* isoforms (Figure 3a), the genetic inhibition of which largely suppresses the longevity of *daf‐2* mutants (Chen et al., 2015; Heimbucher et al., 2015; Kumar et al., 2015; Lin et al., 2018; Riedel et al., 2013). Similarly, *mg54*, a nonsense mutation that disrupts *daf‐16a* and *daf‐16f* (Figure 3a), suppresses the longevity of *daf‐2* mutants (Chen et al., 2015). In contrast, *tm5030* and *tm5032*, which delete parts of the exon specific to *daf‐16a* (Figure 3a), partially suppress the longevity of *daf‐2* mutants, whereas *tm6659*, which deletes *daf‐16f* (Figure 3a), has a small effect on the longevity of *daf‐2* mutants (Chen et al., 2015). We sought to relate transcriptomic changes caused by these seven *daf‐16* mutant alleles to effects on the longevity of *daf‐2* mutants (Figure 3b). *mgDf50* elicited the greatest impact on the transcriptome, and the changes were distinct from those caused by the other *daf‐16* alleles (Figures 2 and 3b). This is likely caused by shorter read length and older version of sequencing machines used for generating *mgDf50* data than data with the other *daf‐16* mutant alleles (Appendix S1). We therefore excluded *mgDf50* as an outlier from our subsequent comparisons among *daf‐16* mutant alleles. The effects of the other *daf‐16* mutant alleles on the transcriptome were positioned along the trajectory formed by *daf‐16* *(* *mgDf47* *)* *;* *daf‐2* and *daf‐2* mutations as follows: *mgDf47* > *mu86* ≈ *mg54* > *tm5030* ≈ *tm5032* > *tm6659* > control (Figure 3b). We also identified strong correlations (absolute *r* = 0.89) between the extents of transcriptomic changes and those of lifespan changes caused by the analyzed six *daf‐16* mutant alleles in *daf‐2* mutants (Tables S1 and S2); *mgDf50* had no corresponding lifespan data (Kumar et al., 2015). Thus, we used *mgDf47* as the basis for further analyses of *daf‐16* mutant alleles because it had the greatest effect on the transcriptome among the six mutant alleles. We determined the overall magnitude of gene expression changes caused by each *daf‐16* mutant allele, which was upregulated or downregulated in *daf‐2* mutants compared to that in *mgDf47* mutants (Figure 3c; Figure S3). We found that the extent of gene expression changes caused by *mu86* and *mg54* was similar to that caused by *mgDf47* (Figure 3c; Figure S3c–e). These data are consistent with the strong allelic nature of these mutations and the marginal contribution of *daf‐16b* to gene expression changes (Kwon et al., 2010). We found that the overall extent of changes caused by *tm5030* and *tm5032* was relatively small compared to that caused by *mgDf47* (Figure 3c; Figure S3f,g). *tm6659* had the smallest impact on the expression of target genes affected by *mgDf47* (Figure 3c; Figure S3h). Together, these results indicate that the extents of lifespan changes caused by these *daf‐16* mutant alleles correlate with the levels of transcriptomic changes in *daf‐2* mutants.

### 3.4 Transcriptomic analysis indicates that *daf‐16*/ *FOXO* mutant alleles exert generally common effects on biological processes in *daf‐2* mutants

To assess the effects of multiple *daf‐16* mutant alleles on the transcriptome of *daf‐2* mutants, we next conducted gene set enrichment analysis (GSEA) (Subramanian et al., 2005) based on WormCat, an annotation database of *C. elegans* genome‐scale data (Higgins et al., 2022; Holdorf et al., 2020), at intermediate and low levels (categories 2 and 3) (Figure 3d; Appendix S5). We commonly detected upregulation of the term “Stress response, Oxidative” in all comparisons. The terms “Stress response, Heavy metal” and “Metabolism, Insulin” were upregulated in *daf‐2* mutants compared to the majority of *daf‐16; daf‐2* mutants except for the animals that contained *tm6659*, the weakest *daf‐16* mutant allele. These data suggest that the *daf‐16f* isoform elicits upregulation of the oxidative stress response but not the response to heavy metal stress or insulin metabolism. Three functionally null alleles, *mgDf50*, *mgDf47*, and *mu86*, tended to downregulate the term “Extracellular material, Collagen”, consistent with the pro‐longevity roles of collagens (Ewald et al., 2015), whose upregulation may require *daf‐16a*, *daf‐16b*, and *daf‐16f*. We also detected common downregulation of the terms “Metabolism, Amino acid” and “Ribosome, Subunit” in *daf‐2* mutants compared with the majority of the analyzed *daf‐16; daf‐2* mutants except for the animals carrying the strongest allele *mgDf50*. These results are consistent with reports indicating that reduced translation and changes in amino acid levels contribute to reduced IIS‐mediated longevity (Depuydt et al., 2013; Edwards et al., 2015). In addition, “Stress response, Pathogen” and “Stress response, C‐type lectin” were downregulated in *daf‐2* mutants compared with the majority of *daf‐16; daf‐2* mutants. These data are consistent with the essential but complex role of immunity in the longevity conferred by upregulation of DAF‐16/FOXO in *daf‐2* mutants (Lee et al., 2021; Park et al., 2021; Podshivalova et al., 2017; Wu et al., 2019).

We additionally conducted GSEA using single cell RNA‐seq data obtained with wild‐type and *daf‐2* mutants (Preston et al., 2019), to analyze the effects of multiple *daf‐16* mutant alleles on the transcriptome of *daf‐2* mutants at a single cell level (Figure 3e). We commonly detected the enrichment of the cell type “001_Sperm” in almost all the comparisons. “013_Cholinergic DA motor neurons” was enriched in the dataset with *tm6659*, but depleted in that with the *tm5030* and *tm5032* alleles, suggesting the specific roles of *daf‐16f* in these neurons for contributing to physiological changes in *daf‐2* mutants. Gene expression in various other cell and tissue types, including FLP neurons, the intestine, hypodermis, pharynx, muscle, seam cells, and germline, was enriched in *daf‐2* mutants compared with the majority of *daf‐16; daf‐2* mutants except for the mutant animals that contained the weakest *tm6659* mutant allele. These data suggest that *daf‐16a* alters gene expression in more diverse cell types in *daf‐2* mutants than *daf‐16b* or *daf‐16f* does. Overall, our transcriptomic analysis revealed the differential effects of individual *daf‐16* mutant alleles on the transcriptome of *daf‐2* mutants at various cell types, which also correlate with phenotypic consequences, including lifespan changes.

### 3.5 Transcriptomic analysis of gene manipulation with respect to four representative longevity transcription factors in the IIS pathway

**FIGURE 4:**
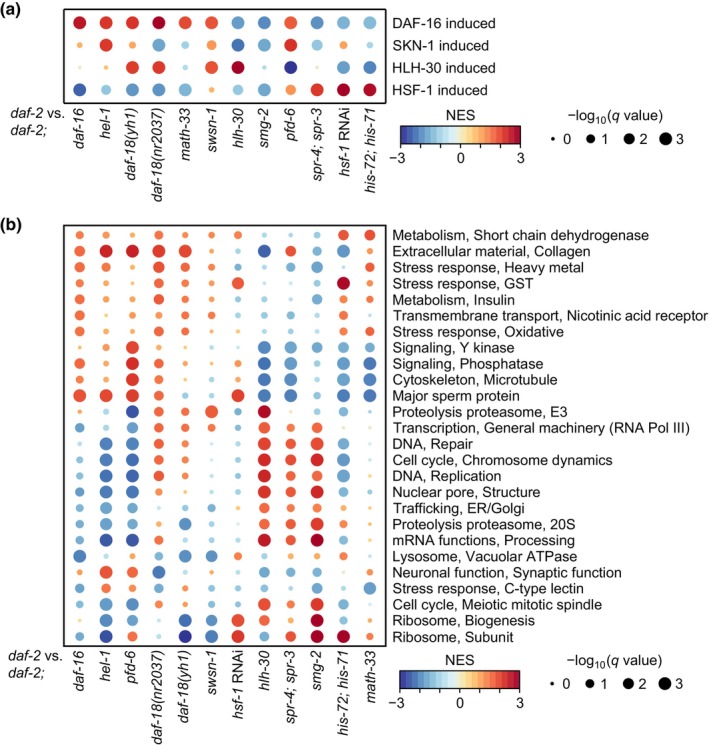
The effects of four representative longevity‐promoting transcription factors that act downstream of *daf‐2* mutants on transcriptomic changes. (a) NES of transcriptomic changes of target genes of representative longevity transcription factors acting in the IIS: DAF16/FOXO (Riedel et al., 2013), SKN‐1/NRF (Ewald et al., 2015), HLH‐30 (Lin et al., 2018), and HSF‐1/HSF1 (Lee et al., 2021). *q*‐values were obtained by calculating the false discovery rate corresponding to each NES. Datasets were sorted based on hierarchical clustering. (b) NES of transcriptomic changes in indicated WormCat terms at intermediate levels by the mutations analyzed in this study. *q*‐values were obtained by calculating the false discovery rate corresponding to each NES. Representative terms were selected based on minimum *q*‐values (*q* = 0.05) among different conditions. The terms were further selected when at least five conditions showed absolute NES greater than 1.5 or at least six conditions showed absolute NES greater than 1.25. Terms were sorted based on hierarchical clustering. NES range from −3 (blue) to +3 (red). The size of black circles correlates with −log_10_(*q*‐value).

To further dissect transcriptional changes caused by key longevity regulators, we analyzed the transcriptome data with *daf‐2* mutants with respect to the target genes of four representative longevity transcription factors acting in the IIS: DAF‐16/FOXO, SKN‐1/nuclear respiratory factor (NRF), HLH‐30/TFEB, and HSF‐1/HSF1 (Altintas et al., 2016; Denzel et al., 2019; Lee & Lee, 2022; Murphy & Hu, 2013). Consistent with our analysis shown in Figure 2, we found that the expression of DAF‐16–induced genes was higher in *daf‐2* mutants than in *hel‐1; daf‐2*, *daf‐2; daf‐18* *(* *yh1* *)*, *daf‐2; daf‐18* *(* *nr2037* *)*, *daf‐2; math‐33*, and *daf‐2; swsn‐1* mutants (Figure 4a). In addition, the expression of DAF‐16‐induced genes was higher in *daf‐2* mutants than in *pfd‐6; daf‐2* mutants. These data are consistent with established relationships between the five factors and DAF‐16 (Gil et al., 1999; Heimbucher et al., 2015; Mihaylova et al., 1999; Ogg & Ruvkun, 1998; Riedel et al., 2013; Seo et al., 2015; Son et al., 2018). Together, HEL‐1, DAF‐18, MATH‐33, SWSN‐1, PFD‐6, and DAF‐16 appear to cooperate for common gene induction.

We also demonstrated that the expression of SKN‐1‐induced genes was higher in *daf‐2* mutants than in *hel‐1; daf‐2* and *pfd‐6; daf‐2* mutants (Figure 4a), suggesting the cooperativity of HEL‐1, PFD‐6, and SKN‐1 in gene induction. Our current analysis recapitulated a previous report (Park et al., 2021) revealing the downregulation of SKN‐1–induced genes in *daf‐2* mutants compared with *daf‐2; daf‐18* *(* *nr2037* *)* mutants but not with *daf‐2; daf‐18* *(* *yh1* *)* mutants (Figure 4a). In addition, many genes that were induced by HLH‐30 in *daf‐2* mutants were suppressed by *daf‐18* *(* *yh1* *)*, *daf‐18* *(* *nr2037* *)*, and *swsn‐1* mutations (Figure 4a), and therefore HLH‐30 appears to cooperate with DAF‐18 and SWSN‐1 in gene induction. This is consistent with previous reports showing the positive relationships between DAF‐18 and DAF‐16 (Gil et al., 1999; Mihaylova et al., 1999; Ogg & Ruvkun, 1998; Park et al., 2021) and between HLH‐30 and DAF‐16 (Lin et al., 2018). We found that the increased expression of HSF‐1–induced genes in *daf‐2* mutants was enriched in genes whose expression was upregulated by PFD‐6 (Figure 4a), which acts downstream of HSF‐1 (Son et al., 2018). Our analysis indicated that the induction of genes by HSF‐1/HSF1 was upregulated by SPR‐3/SPR‐4, and HIS‐71/HIS‐72 as well (Figure 4a), consistent with the role of HSF‐1 in chromatin dynamics (Labbadia & Morimoto, 2015). We also identified unexpected relationships among the four representative longevity‐associated transcription factors. We found that the expression of DAF‐16– and SKN‐1–induced genes was reduced in *daf‐2* mutants compared to that in *daf‐2; hlh‐30* mutants (Figure 4a), raising the possibility of the distinct role of HLH‐30 opposed to DAF‐16 and SKN‐1 in transcriptional regulation. Our analysis also raises the possibility that HSF‐1 is mutually antagonistic to DAF‐16 and HLH‐30 in overall gene induction (Figure 4a). Collectively, these four established longevity transcription factors appear to work with specific subsets of factors in the IIS pathway to mediate the effects of *daf‐2* mutations on physiological processes, including aging.

### 3.6 WormCat analysis of the transcriptomes affected by the inhibition of longevity genes in IIS

To assess the effects of the genetic interventions on the transcriptome of *daf‐2* mutants, we further compared the expression changes of genes associated with WormCat (Higgins et al., 2022; Holdorf et al., 2020) terms at intermediate and low levels (categories 2 and 3) caused by different genetic interventions (Figure 4b; Appendix S6). We were unable to detect universal changes among the comparisons, indicating that these genetic factors contribute to longevity conferred by *daf‐2* mutations in specific ways. Instead, our results indicated that longevity regulators, including HEL‐1, PFD‐6, DAF‐18, SWSN‐1, and MATH‐33, that work with DAF‐16, tended to upregulate the terms “Metabolism, Insulin”, “Metabolism, Short chain dehydrogenase”, “Extracellular material, Collagen”, “Stress response, Heavy metal”, and “Stress response, GST” (Figure 4b). These data are consistent with the important role of metabolism and collagen genes in the longevity conferred by reduced IIS (Dall & Faergeman, 2019; Ewald et al., 2015; Zecic & Braeckman, 2020) and the strong correlation between longevity and stress responses (Epel & Lithgow, 2014; Johnson et al., 2001; Park et al., 2017). Contrarily, HLH‐30, SPR‐3/SPR‐4, and SMG‐2 were associated with upregulation of the terms “Cell cycle, Chromosome dynamics”, “DNA, Repair”, “DNA, Replication”, “Nuclear pore, Structure”, “Trafficking, ER/Golgi”, “Proteolysis proteasome, 20S”, and “mRNA functions, Processing” (Figure 4b). Interestingly, the term “Ribosome, Subunit” was upregulated in six cases but downregulated in five cases in *daf‐2* mutants (Figure 4b). These results are consistent with previous reports suggesting that ribosomes can positively or negatively contribute to aging and longevity in a context‐dependent manner (Depuydt et al., 2013; Walther et al., 2015). Together these data suggest that the majority of different genetic interventions affect certain signaling pathways to suppress longevity conferred by *daf‐2* mutations.

### 3.7 Analysis of intron‐derived and non‐coding RNAs

**FIGURE 5:**
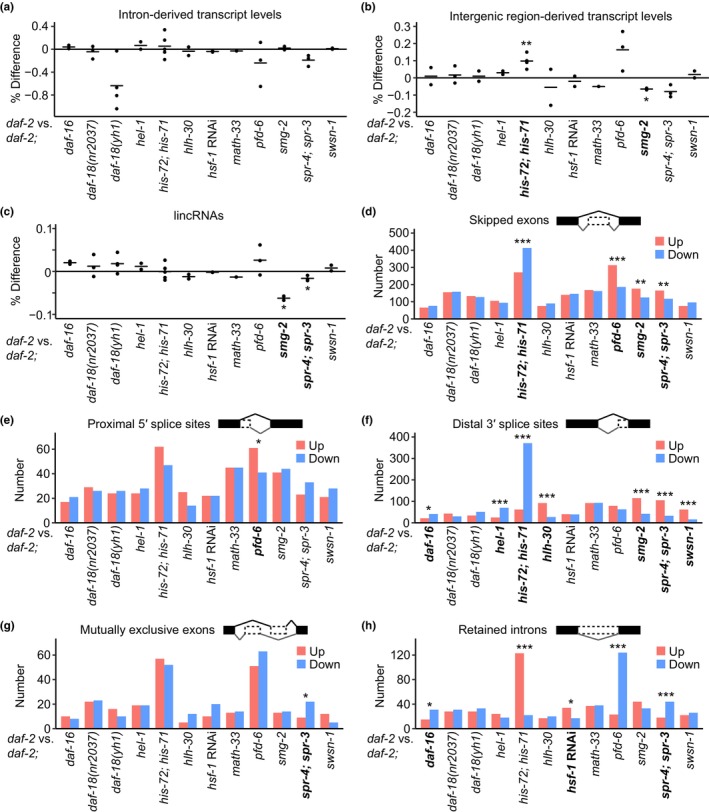
Analysis of representative non‐coding RNAs and splicing of mRNAs affected by genetic inhibitions that suppress the longevity of *daf‐2* mutants. (a–c) Overall changes in the levels of intron‐derived transcripts (a), intergenic region‐derived transcripts (b), and long intergenic noncoding RNA (lincRNA) (c) by mutations in the longevity‐promoting genetic factors analyzed in this study. *p* value of two‐tailed Welch's *t* test is shown on top of each panel, *p < 0.05, **p < 0.01, ***p < 0.001. Genes whose mutations elicited significant changes are indicated in bold in this figure. (d–h) The number of splicing events, including skipped exons (d), proximal 5′ splice sites (e), distal 3′ splice sites (f), mutually exclusive exons (usage of distal exons) (g), and retained introns (h), which were upregulated or downregulated by the mutations analyzed in this study. *p* values of two‐tailed Fisher's exact test for counts are shown on top of each panel, *p < 0.05, **p < 0.01, ***p < 0.001.

In our previous report regarding systematic transcriptome analysis for identifying aging biomarkers using wild‐type and *daf‐2* mutants, we revealed that intron‐ and intergenic region‐derived transcripts, noncoding RNAs, and the usage of distal 3′ splice sites in mRNA transcripts are upregulated during aging (Ham et al., 2022). We therefore determined whether these biomarkers of aging were affected by the genetic inhibition of each of the 11 genetic factors, which decreases the longevity of *daf‐2* mutants. However, none of the genetic inhibitions significantly affected intron‐derived transcripts (Figure 5a). In contrast, intergenic region‐derived transcript levels were increased in *daf‐2* mutants compared with *daf‐2 his‐72; his‐71* mutants but were decreased in *daf‐2* mutants compared with *smg‐2; daf‐2* mutants (Figure 5b). These data suggest that HIS‐71 and HIS‐72 regulate the transcription of non‐annotated genes. In addition, SMG‐2 may contribute to longevity conferred by *daf‐2* mutations by suppressing the expression of intergenic region‐derived transcripts. Additionally, the levels of long intergenic noncoding RNAs (lincRNAs) were lower in *daf‐2* mutants than in *smg‐2; daf‐2* and *spr‐4; daf‐2; spr‐3* mutants (Figure 5c). These data raise the possibility that SMG‐2 and REST contribute to longevity in *daf‐2* mutants by suppressing the induction of lincRNAs.

We next analyzed the changes of alternative splicing caused by genetic inhibition of each of the 11 factors in *daf‐2* mutants. Among them we found that *his‐72; his‐71* mutations elicited the greatest impact on alternative splicing. Specifically, *his‐72; his‐71* increased the levels of skipped exons and the usage of distal 3′ splice sites in *daf‐2* mutants while decreasing those of retained introns (Figure 5d,f,h). These data suggest that HIS‐71/HIS‐72 inhibits the usage of distal 3′ splice sites to promote longevity in *daf‐2* mutants. Different from the effects of *his‐72; his‐71* mutations, *pfd‐6*, *smg‐2*, and *spr‐4* *;* *spr‐3* mutations decreased the levels of skipped exons (Figure 5d). In addition, *pfd‐6* mutations decreased the usage of proximal 5′ splice sites and increased the levels of retained introns (Figure 5e,h). *smg‐2* mutations decreased the usage of distal 3′ splice sites (Figure 5f). *spr‐4; spr‐3* mutations also decreased the usage of distal 3′ splice sites but increased the levels of retained introns (Figure 5f). These data imply distinct roles of HIS‐71/HIS‐72, PFD‐6, SMG‐2, and SPR‐3/SPR‐4 in alternative splicing. Overall, our data suggest that different genetic interventions affect specific subsets of structural and functional elements of RNAs to mediate the effects of *daf‐2* mutations on aging.

### 3.8 Several longevity regulators that were identified with our transcriptome analysis act together to promote the longevity of *daf‐2* mutants

**FIGURE 6:**
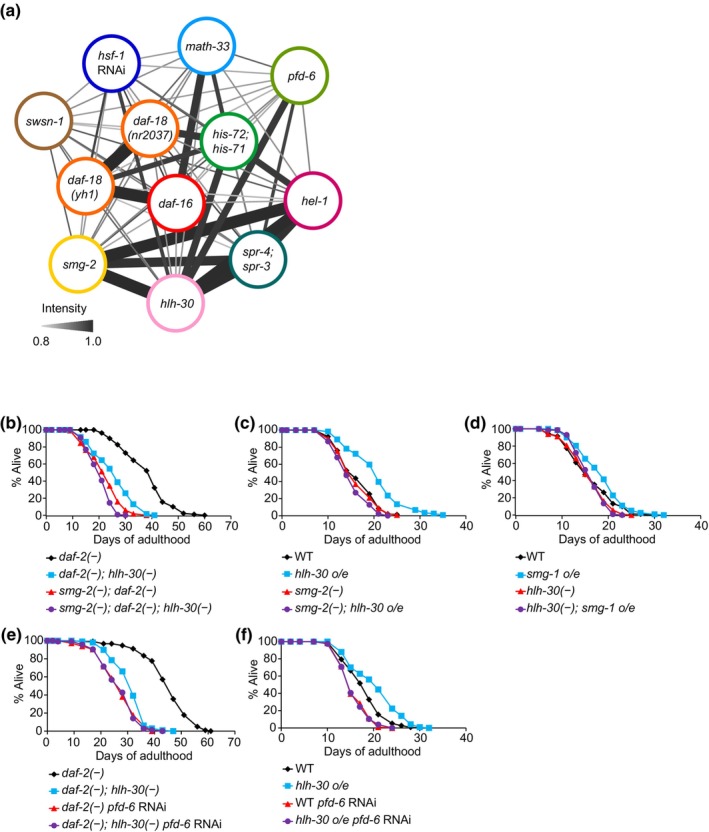
SMG‐2/UPF1, HLH‐30/TFEB, and PFD‐6/PFDN6 act coordinately to promote longevity caused by *daf‐2* mutations. (a) A network of various longevity factors reconstructed by using a consensus approach based on correlation coefficients of transcriptome data. The widths and darkness of lines that connect datasets correlate with the intensities of the links from 0.8 (gray) to 1 (black). Links with intensities ≥0.8 are shown. See Appendix S7 for detailed values of the links. (b) Lifespan curves of *daf‐2* *(* *e1370* *)* [*daf‐2* *(−)*], *daf‐2* *(−)* *;* *hlh‐30* *(* *tm1978* *)* [*hlh‐30* *(−)*], *smg‐2* *(* *qd101* *)* [*smg‐2* *(−)*]*;* *daf‐2* *(−)*, and *smg‐2* *(−)* *;* *daf‐2* *(−)* *;* *hlh‐30* *(−)* animals. (c) The lifespan of wild‐type (WT), *smg‐2* *(−)*, *hlh‐30* overexpressing (*o/e*) (*sqIs17* *[* *hlh‐30p::hlh‐30::GFP; rol‐6* *(* *su1006* *)]*), and *smg‐2* *(−)* *;* *hlh‐30 o/e* animals. (d) The lifespan of WT, *hlh‐30* *(−)*, *smg‐1 o/e* (*yhEx330* *[* *smg‐1p::smg‐1::gfp* *;* *odr‐1p::RFP* *]*), and *hlh‐30* *(−)* *;* *smg‐1 o/e* animals. (e) Lifespan curves of *daf‐2* *(−)* and *daf‐2* *(−)* *;* *hlh‐30* *(−)*animals treated with *pfd‐6* RNAi. (f) Lifespan of WT and *hlh‐30 o/e* animals treated with *pfd‐6* RNAi. At least 75 animals were used for each condition of lifespan assays. See Table S3 for additional repeats and statistical analysis of the lifespan data shown in this figure.

We sought to identify novel genetic interactions between the pairs of longevity regulators acting downstream of DAF‐2/insulin/IGF‐1 receptor. We first functionally analyzed the relationship between *hlh‐30*/*TFEB* and *smg‐2*/*UPF1*, as mutations in these genes induced transcriptomic changes with the highest similarity among previously uncharacterized interactions in the *daf‐2* mutant background (interaction intensity = 0.96) (Figure 6a; Figure S4; Appendices S7 and S8). In addition, we analyzed the interaction between *hlh‐30*/*TFEB* and *pfd‐6*/*PFDN6* because *pfd‐6* have a higher similarity with *hlh‐30* (interaction intensity = 0.89) than *daf‐16* (interaction intensity = 0.19); DAF‐16 activity as a transcription factor was enhanced by PFD‐6 (Son et al., 2018) (Figure 6a; Figure S5; Appendices S7 and S9). We demonstrated that *hlh‐30; smg‐2* double mutations had slightly stronger suppressive effects on the longevity of *daf‐2* mutants than the single mutations (Figure 6b). These data suggest that SMG‐2/UPF1 and HLH‐30/TFEB regulate common targets, but the extent of regulation by each factor is partially additive. Importantly, we found that the long lifespan conferred by *hlh‐30* overexpression (Lapierre et al., 2013; Lin et al., 2018) was suppressed by *smg‐2* mutations (Figure 6c). Conversely, longevity conferred by the overexpression of SMG‐1 (Son et al., 2017), which phosphorylates and activates SMG‐2/UPF1 to enhance nonsense‐mediated mRNA decay (NMD) (Kim & Maquat, 2019; Kwon et al., 2023; Son & Lee, 2017), was suppressed by *hlh‐30* mutations (Figure 6d). Therefore, *hlh‐30* and *smg‐2* act on substantially overlapping transcriptomic changes and promote longevity in concert. We also demonstrated that *hlh‐30* mutations did not further shorten the lifespan of *daf‐2* mutants treated with *pfd‐6* RNAi (Figure 6e). Moreover, we found that the long lifespan conferred by *hlh‐30* overexpression (Lapierre et al., 2013; Lin et al., 2018) was suppressed by *pfd‐6* mutation (Figure 6f). Thus, *hlh‐30* and *pfd‐6* also appear to promote longevity together by regulating common target genes. These data raise the possibility of unexpected collaboration between these longevity regulators to delay aging and to confer longevity.

## 4 DISCUSSION

Transcriptomic analysis is useful for identifying novel and multiple genetic interactions that elicit phenotypic consequences. In the current work, we analyzed RNA‐seq data featuring the genetic inhibition of various aging‐regulating factors that contribute to longevity conferred by *daf‐2* mutations in *C. elegans*. Our transcriptomic analysis verified established genetic interactions and unraveled the possibilities of unexpected epistatic relationships of downstream components in the IIS pathway. Our results also indicate that the extent of lifespan changes caused by various *daf‐16* mutant alleles correlates with the extent of transcriptomic changes in *daf‐2* mutants. In addition, different genetic interventions appear to affect specific subsets of structural and functional RNA elements in *daf‐2* mutants. Lastly, we identified genetic interactions between longevity regulators, *hlh‐30* and *smg‐2*, and *pfd‐6* and *hlh‐30*, which cause common transcriptomic changes for mediating longevity conferred by *daf‐2* mutations in concert. Overall, our combinatorial transcriptomic analysis proved useful for determining novel and multiple genetic interactions and for overcoming the limitations of phenotype‐based classical genetic approaches.

Our unbiased analysis revealed the unexpected actions regarding longevity regulators in *daf‐2* mutants. Different from a previous study suggesting the role of MATH‐33 in the stability of DAF‐16 through deubiquitination (Heimbucher et al., 2015), our finding implies that MATH‐33 downregulates DAF‐2. The quality‐control ubiquitin ligase CHIP triggers the turnover of DAF‐2 for affecting IIS and consequently lifespan (Tawo et al., 2017). The stability of CHIP is affected by other ubiquitin ligases and deubiquitylation enzymes (Hohfeld & Hoppe, 2018). Thus, these studies and our current work raise the possibility that MATH‐33 contributes to the stability of CHIP that downregulates DAF‐2. In addition, our results suggested that HIS‐71/HIS‐72, SPR‐3/SPR‐4, and SMG‐2 act with DAF‐2 in the same pathway and that HEL‐1 upregulates DAF‐2 via feedforward signaling. These data support the requirement of multifaceted and comprehensive analysis to properly understand longevity regulators in the IIS pathway.

Our analysis suggests two distinct groups of longevity regulators in *daf‐2* mutants. First, mutations in *daf‐18*, *math‐33*, *swsn‐1*, or *hel‐1* elicited similar transcriptomic changes caused by *daf‐16* mutations in *daf‐2* mutants, supporting genetic relationships established by classical genetic studies, including lifespan assays (Gems et al., 1998; Gil et al., 1999; Heimbucher et al., 2015; Mihaylova et al., 1999; Ogg & Ruvkun, 1998; Riedel et al., 2013; Seo et al., 2015). In contrast, mutations in *hlh‐30*, *spr‐4; spr‐3*, and *smg‐2* elicited distinguishable transcriptomic changes compared to those caused by *daf‐16* mutations in *daf‐2* mutants. We also noticed that 77% of genes differentially regulated by HLH‐30 did not overlap with those regulated by DAF‐16 in *daf‐2* mutants, consistent with context‐dependent cooperative and antagonistic regulation between DAF‐16 and HLH‐30 (Mony et al., 2021). Additionally, we noted that only 12% of genes that were downregulated by *spr‐4; spr‐3* mutations in *daf‐2* mutants at Day 10 of adulthood overlapped with class I DAF‐16 target genes, which are defined as genes upregulated in *daf‐2* mutants in a DAF‐16‐dependent manner (Murphy et al., 2003). We noted that the roles of PFD‐6 is entangled with different regulators. PFD‐6 appears to work with SKN‐1 and HSF‐1 as well as DAF‐16. In fact, 66% of genes that were downregulated by *pfd‐6* mutations did not overlap with those downregulated by *daf‐16* mutations in *daf‐2* mutants (Son et al., 2018). Our data also raise another possibility that *pfd‐6* and *hlh‐30* act on substantially overlapping transcriptomic changes and promote longevity in concert. Overall, these data suggest that the established proteins crucial for the longevity of *daf‐2* mutants play distinct roles in the regulation of DAF‐16.

In addition to the mRNA analysis, our work demonstrated that different genetic interventions affected specific subsets of intron‐derived or non‐coding RNAs in *daf‐2* mutants. However, because of the technical limitations of publicly available RNA‐seq data, we were not able to analyze non‐coding RNAs in depth or to examine whether genetic factors whose depletion decreases longevity caused by *daf‐2* mutations contribute to universal changes in non‐coding RNA levels. Future research is required to overcome these technical constraints by employing advanced sequencing technologies or generating targeted datasets.

Our transcriptomic and functional analyses established the importance of previously unrecognized genetic interactions between HLH‐30 and SMG‐2 for longevity in *daf‐2* mutants. HLH‐30 is important for autophagy, a key protein surveillance system (Dall & Faergeman, 2019; Denzel et al., 2019; Lapierre et al., 2013; O'Rourke & Ruvkun, 2013), and SMG‐2 is the crucial component of NMD, an mRNA quality control system (Kim & Maquat, 2019; Kwon et al., 2023; Son & Lee, 2017). NMD recognizes premature termination codons that potentially generate truncated abnormal and/or misfolded polypeptides (Goetz & Wilkinson, 2017; Kim & Maquat, 2019; Son & Lee, 2017). These truncated polypeptides are eliminated by proteostasis systems, including autophagy (Aman et al., 2021; Lamark & Johansen, 2012; Steffen & Dillin, 2016). Thus, the NMD‐mediated mRNA surveillance and autophagy‐mediated proteostasis systems appear to be closely associated in the promotion of cellular homeostasis, contributing to longevity. It will be interesting to determine the mechanisms by which NMD and autophagy interact to mediate longevity in future research.

We analyzed RNA‐seq data obtained from animals with genetic inhibition of factors crucial for longevity attributable to *daf‐2* mutations in *C. elegans*. However, transcriptomic changes caused by the genetic inhibition of these factors are possibly associated with altered responses to heat shock and oxidative stress and/or dauer formation independently of longevity. In addition, dissecting the difference between chronological and physiological ages at the transcriptomic level is required to understand the mechanisms underlying each of these physiological processes (Ham et al., 2022). Furthermore, because we analyzed RNA‐seq data obtained from whole bodies, we should be cautious about potential caveats, including tissue heterogeneity, signal dilution, and biological noise. Notably, *daf‐2* mutations contribute to longevity by affecting particular longevity regulators in a tissue‐specific manner (Kaletsky et al., 2016; Libina et al., 2003; Roy et al., 2022; Zhao et al., 2021). Tissue‐specific analysis remains essential for a nuanced interpretation of the molecular mechanisms underlying longevity conferred by *daf‐2* mutations. Importantly, transcriptomic analysis reveals correlations in gene expression patterns, but does not directly prove causation. Therefore, identified relationships between genes in a pathway should be further validated using other experimental techniques, such as genetic manipulations and functional physiologic assays. To identify transcriptomic changes specifically associated with longevity and their causality, future research aiming at integrative analysis using other omics data obtained using ChIP‐seq, CLIP‐seq, single cell RNA‐seq (Hwang, 2023; Kim, 2023; Kim & Lee, 2023; Ryu et al., 2023), and mass spectrometry will be necessary.

In conclusion, our work suggests that comprehensive transcriptomic analysis is useful for identifying and quantifying genetic interactions in complex physiological processes, including aging. Our approaches have the potential to facilitate transcriptomic analyses of other genetic interventions that contribute to longevity, such as reduced mTOR signaling and mild inhibition of mitochondrial respiration in *C. elegans* and other model organisms. Moreover, our work can serve as a guideline for the future application of analyses of transcriptomic changes to provide insights into aging and age‐associated diseases in humans.

## AUTHOR CONTRIBUTIONS

**Seokjin Ham**: Conceptualization; data curation; formal analysis; investigation; visualization; methodology; writing—original draft. **Sieun S. Kim**: Formal analysis; validation; methodology; writing—original draft. **Sangsoon Park**: Formal analysis; validation. **Hyunwoo C. Kwon**: Formal analysis; validation. **Seokjun G. Ha**: Formal analysis; validation. **Yunkyu Bae**: Data curation; writing—original draft. **Gee‐Yoon Lee**: Investigation. **Seung‐Jae V. Lee**: Conceptualization; supervision; funding acquisition; investigation; writing—original draft; project administration.

## FUNDING INFORMATION

This work was supported by the National Research Foundation of Korea (NRF) grant funded by the Korea government (MSIT) NRF‐2019R1A3B2067745 to Seung‐Jae V. Lee.

## CONFLICT OF INTEREST STATEMENT

The authors declare that they have no competing interests.

## Supporting information


Figure S1.

Table S1.

Table S2.

Table S3.

Appendix S1.

Appendix S2.

Appendix S3.

Appendix S4.

Appendix S5.

Appendix S6.

Appendix S7.

Appendix S8.

Appendix S9.

## Data Availability

The published sequencing data used in this study (Appendix S1) are available from the Sequence Read Archive (SRA) and the Gene Expression Omnibus (GEO) in the National Center for Biotechnology Information (NCBI).
