# Supplementary material for: Combinatorial transcriptomic and genetic dissection of insulin/IGF‐1 signaling‐regulated longevity in Caenorhabditis elegans
Source: Aging Cell. 2024 Mar 26;23(7):e14151. doi: 10.1111/acel.14151 (PMC11258480; doi:10.1111/acel.14151)
Supplement: Supplementary file 1 — Figure S1. [file ACEL-23-e14151-s005.pdf]

Figure S1

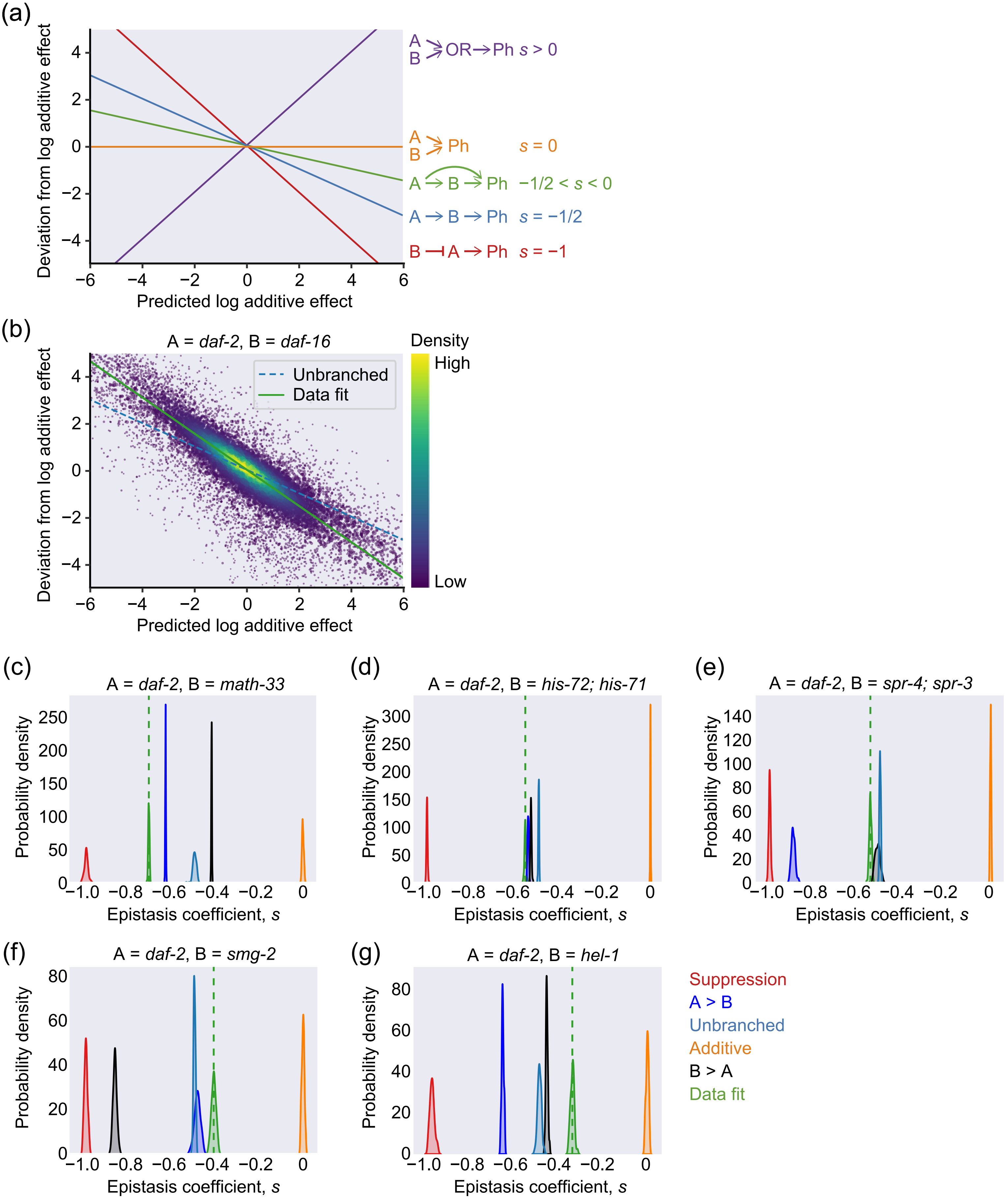

(a)
Deviation from log additive effect
Predicted log additive effect
A → B → OR → Ph s > 0
A, B → Ph s = 0
A → B → Ph −1/2 < s < 0
A → B → Ph s = −1/2
B ⊣ A → Ph s = −1
(b)
A = daf-2, B = daf-16
Unbranched
Data fit
Density
High
Low
Deviation from log additive effect
Predicted log additive effect
(c)
A = daf-2, B = math-33
(d)
A = daf-2, B = his-72; his-71
(e)
A = daf-2, B = spr-4; spr-3
(f)
A = daf-2, B = smg-2
(g)
A = daf-2, B = hel-1
Probability density
Epistasis coefficient, s
Suppression
A > B
Unbranched
Additive
B > A
Data fit

Figure S2

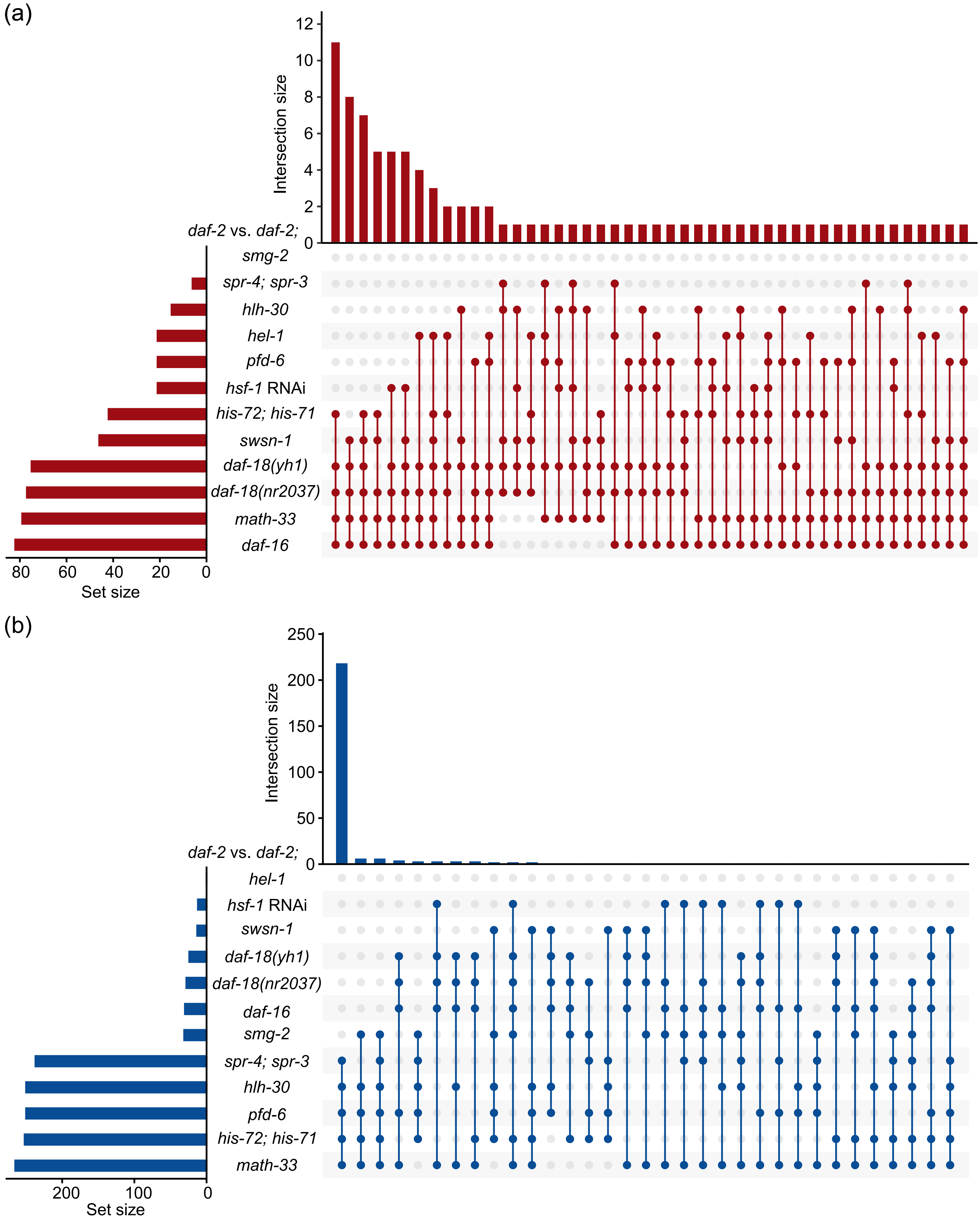
(a)
Intersection size
daf-2 vs. daf-2;
smg-2
spr-4; spr-3
hlh-30
hel-1
pfd-6
hsf-1 RNAi
his-72; his-71
swsn-1
daf-18(yh1)
daf-18(nr2037)
math-33
daf-16
Set size
(b)
Intersection size
daf-2 vs. daf-2;
hel-1
hsf-1 RNAi
swsn-1
daf-18(yh1)
daf-18(nr2037)
daf-16
smg-2
spr-4; spr-3
hlh-30
pfd-6
his-72; his-71
math-33
Set size

Figure S3

(a)

mgDf50 *daf-2* vs *daf-16(mgDf50); daf-2*

mgDf47 *daf-2* vs *daf-16(mgDf47); daf-2*

mu86 *daf-2* vs *daf-16(mu86); daf-2*

mg54 *daf-2* vs *daf-16(mg54); daf-2*

tm5030 *daf-2* vs *daf-16(tm5030); daf-2*

tm5032 *daf-2* vs *daf-16(tm5032); daf-2*

tm6659 *daf-2* vs *daf-16(tm6659); daf-2*

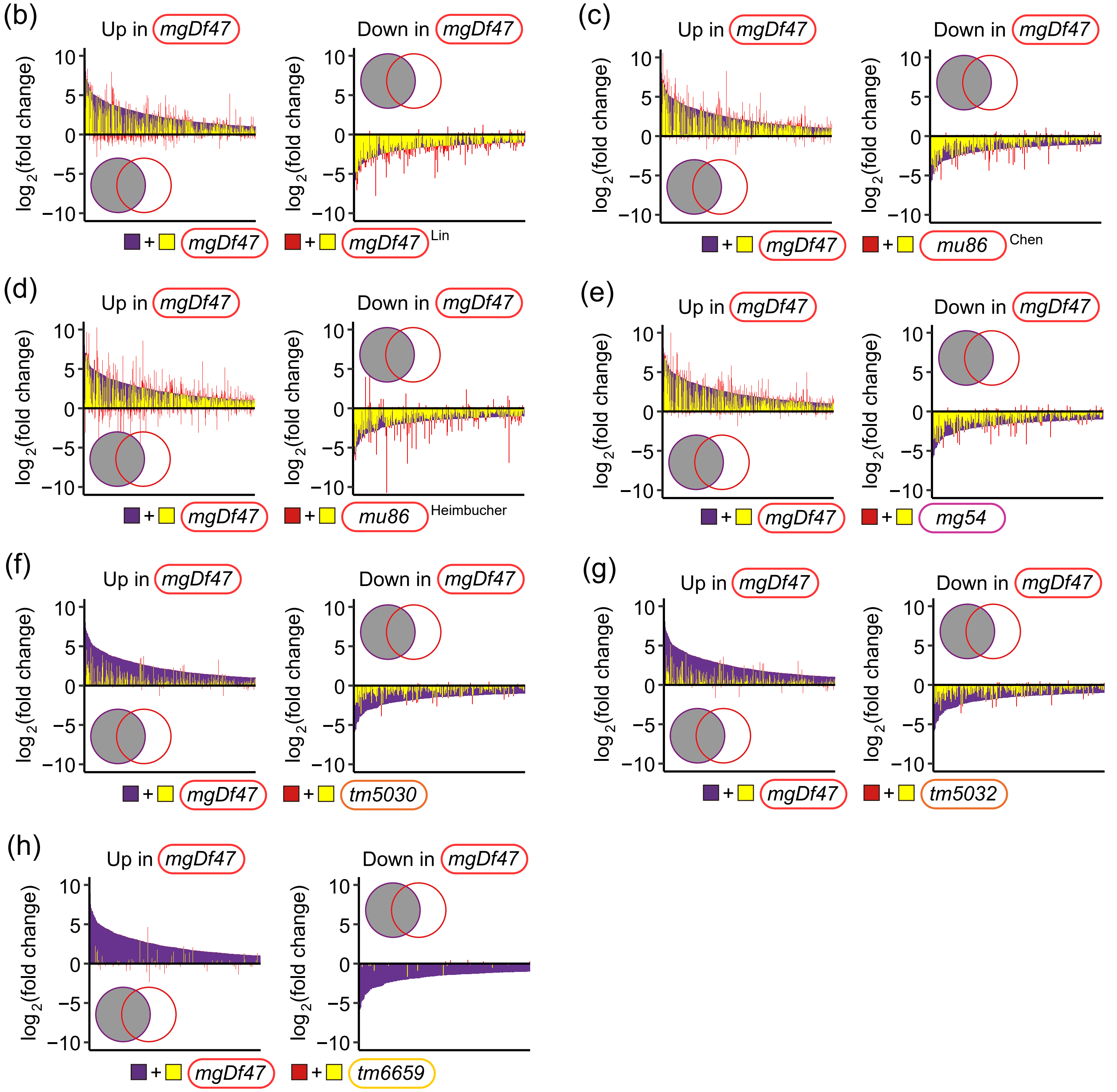

Figure S4

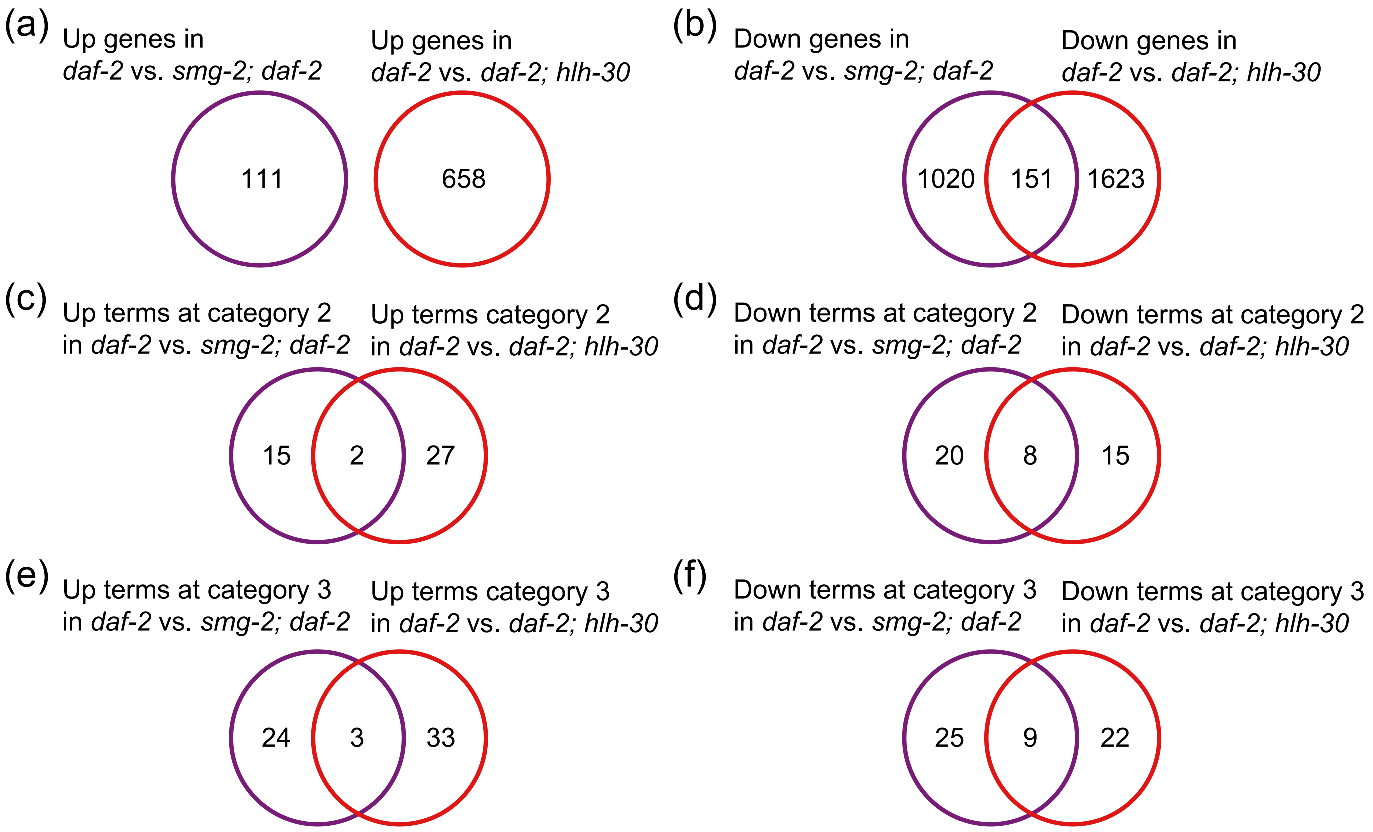

(a)
Up genes in
daf-2 vs. *smg-2; daf-2*
Up genes in
daf-2 vs. *daf-2; hlh-30*
111
658
(b)
Down genes in
daf-2 vs. *smg-2; daf-2*
Down genes in
daf-2 vs. *daf-2; hlh-30*
1020
151
1623
(c)
Up terms at category 2
in *daf-2* vs. *smg-2; daf-2*
Up terms category 2
in *daf-2* vs. *daf-2; hlh-30*
15
2
27
(d)
Down terms at category 2
in *daf-2* vs. *smg-2; daf-2*
Down terms at category 2
in *daf-2* vs. *daf-2; hlh-30*
20
8
15
(e)
Up terms at category 3
in *daf-2* vs. *smg-2; daf-2*
Up terms category 3
in *daf-2* vs. *daf-2; hlh-30*
24
3
33
(f)
Down terms at category 3
in *daf-2* vs. *smg-2; daf-2*
Down terms at category 3
in *daf-2* vs. *daf-2; hlh-30*
25
9
22

Figure S5

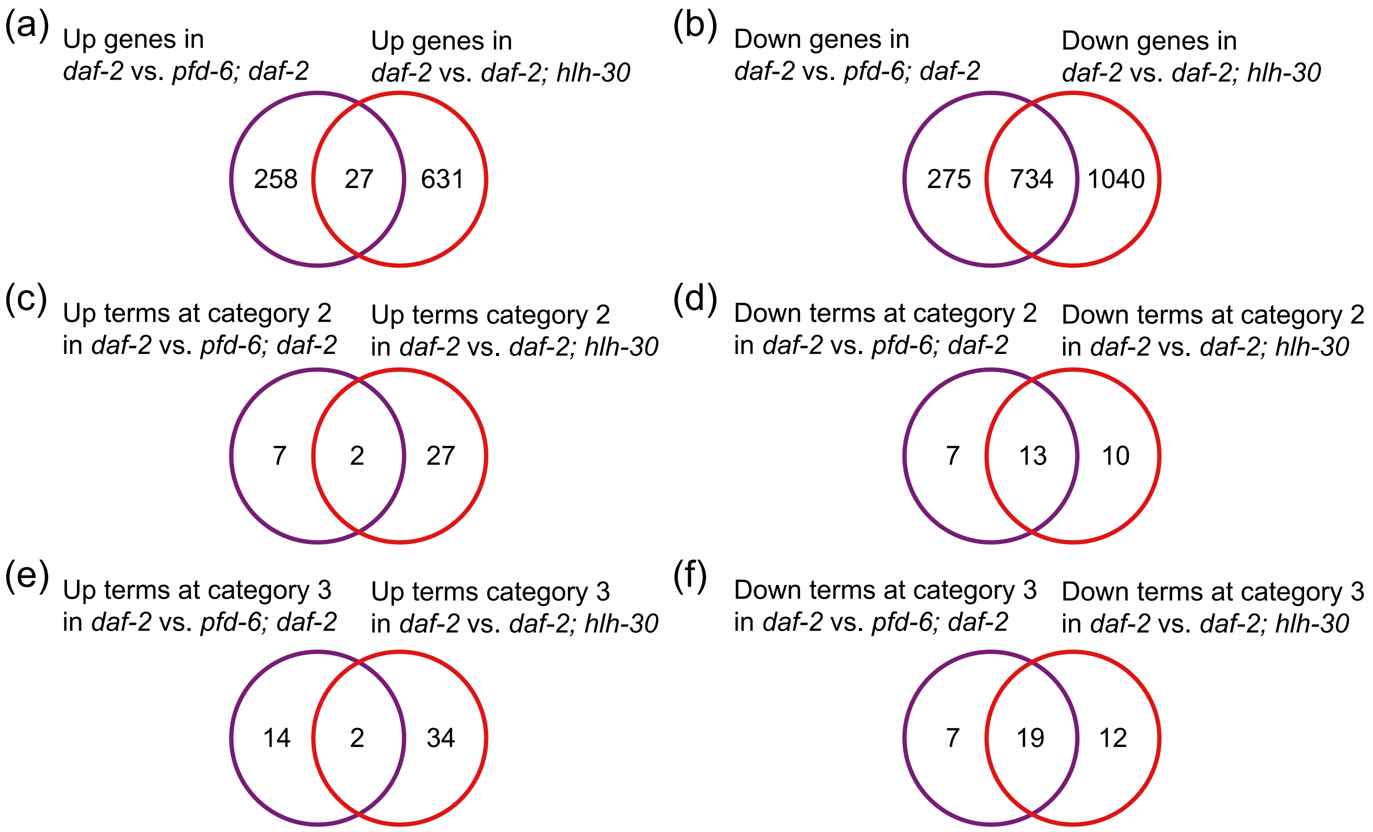
(a)
Up genes in
daf-2 vs. pfd-6; daf-2
Up genes in
daf-2 vs. daf-2; hlh-30
258
27
631
(b)
Down genes in
daf-2 vs. pfd-6; daf-2
Down genes in
daf-2 vs. daf-2; hlh-30
275
734
1040
(c)
Up terms at category 2
in daf-2 vs. pfd-6; daf-2
Up terms category 2
in daf-2 vs. daf-2; hlh-30
7
2
27
(d)
Down terms at category 2
in daf-2 vs. pfd-6; daf-2
Down terms at category 2
in daf-2 vs. daf-2; hlh-30
7
13
10
(e)
Up terms at category 3
in daf-2 vs. pfd-6; daf-2
Up terms category 3
in daf-2 vs. daf-2; hlh-30
14
2
34
(f)
Down terms at category 3
in daf-2 vs. pfd-6; daf-2
Down terms at category 3
in daf-2 vs. daf-2; hlh-30
7
19
12
