# Supplementary material for: Combinatorial transcriptomic and genetic dissection of insulin/IGF‐1 signaling‐regulated longevity in Caenorhabditis elegans
Source: Aging Cell. 2024 Mar 26;23(7):e14151. doi: 10.1111/acel.14151 (PMC11258480; doi:10.1111/acel.14151)
Supplement: Supplementary file 2 — Table S1. [file ACEL-23-e14151-s008.docx]

**Table S1. The extents of transcriptomic changes and those of lifespan changes caused by various *daf-16* mutant alleles in *daf-2* mutants. *daf-16* mutant alleles from different sources: ^Riedel^*mgDf47* (Riedel et al., 2013), ^Lin^*mgDf47* (Lin et al., 2018), ^Chen^*mu86* (Chen et al., 2015), and ^Heimbucher^*mu86* (Heimbucher et al., 2015).**

| Mutations | RNA-seq replicate | Relative distance to *daf-2* mutant samples | Lifespan sample replicate | % change mean survival compared to *daf-2* mutants |
| --- | --- | --- | --- | --- |
| *daf-16(mgDf50)* | 1 | 2.58 | No data | No data |
|  | 2 | 2.47 |  |  |
| *daf-16 (^Riedel^mgDf47)* | 1 | 3.94 |  |  |
|  | 2 | 4.27 |  |  |
|  | Mean | 4.11 | Single | -72.7 |
| *daf-16(^Lin^mgDf47)* | 1 | 2.11 | 1 | -69.8 |
|  | 2 | 1.84 | 2 | -62.1 |
| *daf-16(^Chen^mu86)* | 1 | 2.45 | 1 | -66.0 |
|  | 2 | 2.34 | 2 | -68.0 |
|  | 3 | 2.00 | 3 | -67.0 |
| *daf-16 (^Heimbucher^mu86)* |  |  | 1 | -73.5 |
|  |  |  | 2 | -68.3 |
|  | Single | 2.04 | Mean | -70.9 |
| *daf-16(mg54)* | 1 | 2.31 | 1 | -65.0 |
|  | 2 | 2.39 | 2 | -67.0 |
|  | 3 | 1.91 | 3 | -64.0 |
| *daf-16(tm5030)* | 1 | 1.18 | 1 | -18.0 |
|  | 2 | 1.20 | 2 | -17.0 |
|  | 3 | 0.74 | 3 | -14.0 |
| *daf-16(tm5032)* | 1 | 1.17 | 1 | -17.0 |
|  | 2 | 1.13 | 2 | -22.0 |
|  | 3 | 0.86 | 3 | -23.0 |
| *daf-16(tm6659)* | 1 | 0.23 | 1 | 0.0 |
|  | 2 | 0.18 | 2 | 0.0 |
|  | 3 | 0.19 | 3 | 0.0 |
|  | Correlation coefficient, *r* | -0.89 | *p* | 3.42E-07 |
