# Supplementary material for: Combinatorial transcriptomic and genetic dissection of insulin/IGF‐1 signaling‐regulated longevity in Caenorhabditis elegans
Source: Aging Cell. 2024 Mar 26;23(7):e14151. doi: 10.1111/acel.14151 (PMC11258480; doi:10.1111/acel.14151)
Supplement: Supplementary file 3 — Table S2. [file ACEL-23-e14151-s007.docx]

**Table S2. The extents of transcriptomic changes and those of lifespan changes caused by specific genetic inhibition that suppresses longevity caused by *daf-2* mutations. Dataset of *daf-16* mutants was obtained from the data with *daf-16(mgDf47)* (Riedel et al., 2013).**

| Mutations | RNA-seq replicate | Relative distance to *daf-2* mutant samples | Lifespan sample replicate | % change mean survival compared to *daf-2* mutants |
| --- | --- | --- | --- | --- |
| *daf-16(mgDf47)* | 1 | 3.22 |  |  |
|  | 2 | 3.21 |  |  |
|  | Mean | 3.21 | Single | -72.7 |
| *hel-1(gk148684)* | 1 | 0.58 | 1 | -42.5 |
|  | 2 | 0.81 | 2 | -39.2 |
|  | Mean | 0.70 | Mean | -40.9 |
| *his-72(tm2066); his-71(ok2289)* | 1 | 5.72 | 1 | -28.6 |
|  | 2 | 5.80 | 2 | -31.7 |
|  | 3 | 5.71 | 3 | -20.4 |
|  | 4 | 5.92 |  |  |
|  | 5 | 5.82 |  |  |
|  | Mean | 5.79 | Mean | -26.9 |
| *hlh-30(tm1978lf)* | 1 | 0.39 | 1 | -55.2 |
|  | 2 | 0.93 | 2 | -39.2 |
|  | Mean | 0.66 | Mean | -47.2 |
| *hsf-1 RNAi* | 1 | 0.39 | No data | No data |
|  | 2 | 0.53 |  |  |
|  | Mean | 0.46 |  |  |
| *math-33(tm3561)* |  |  | 1 | -73.7 |
|  |  |  | 2 | -69.0 |
|  | Single | 1.24 | Mean | -71.3 |
| *pfd-6(gk493446)* | 1 | 0.46 | 1 | -30.8 |
|  | 2 | 0.66 | 2 | -32.7 |
|  | 3 | 0.93 |  |  |
|  | Mean | 0.68 | Mean | -31.7 |
| *smg-2(qd101)* | 1 | 1.95 | 1 | -46.9 |
|  | 2 | 0.66 | 2 | -19.4 |
|  |  |  | 3 | -37.7 |
|  |  |  | 4 | -45.8 |
|  |  |  | 5 | -54.7 |
|  | Mean | 1.30 | Mean | -40.9 |
| *spr-4(by105); spr-3(ok2525)* | 1 | 0.45 | 1 | -34.2 |
|  | 2 | 0.55 | 2 | -23.6 |
|  | 3 | 0.64 | 3 | -35.2 |
|  |  |  | 4 | -22.8 |
|  |  |  | 5 | -27.5 |
|  | Mean | 0.55 | Mean | -28.6 |
| *swsn-1(os22ts)* | 1 | 1.28 |  |  |
|  | 2 | 1.31 |  |  |
|  | Mean | 1.30 | Single | -58.6 |
|  | Correlation coefficient, *r* | 0.13 | *p* | 0.73 |
