# Supplementary material for: Combinatorial transcriptomic and genetic dissection of insulin/IGF‐1 signaling‐regulated longevity in Caenorhabditis elegans
Source: Aging Cell. 2024 Mar 26;23(7):e14151. doi: 10.1111/acel.14151 (PMC11258480; doi:10.1111/acel.14151)
Supplement: Supplementary file 4 — Table S3. [file ACEL-23-e14151-s003.docx]

**Table S3. Statistical analysis of lifespan assay data and additional repeats.**

| **Strain** | **Mean lifespan** ± **s.e.m. (days)** | **75th percentile** | **% lifespan change** | **Number of animals that died/total** | ***p* value vs. control** | **Figure in text** |
| --- | --- | --- | --- | --- | --- | --- |
| *daf-2(e1370)* | 38.17 ±0.87 | 44 | - | 96/120 | - | Fig. 6b |
| *smg-2(qd101); daf-2(e1370)* | 22.30 ±0.57 | 27 | -41.58% | 120/150 | <0.0001 | Fig. 6b |
| *daf-2(e1370); hlh-30(tm1978)* | 25.79 ±0.66 | 33 | -32.43% | 137/150 | <0.0001 | Fig. 6b |
| *smg-2(qd101); daf-2(e1370); hlh-30(tm1978)* | 20.59 ±0.38 | 24 | -46.06% | 138/150 | <0.0001 | Fig. 6b |
| *daf-2(e1370)* | 45.05 ±1.23 | 57 | - | 141/150 | - | - |
| *smg-2(qd101); daf-2(e1370)* | 26.64 ±1.08 | 33 | -40.87% | 126/150 | <0.0001 | - |
| *daf-2(e1370); hlh-30(tm1978)* | 28.68 ±0.68 | 35 | -36.34% | 147/150 | <0.0001 | - |
| *smg-2(qd101); daf-2(e1370); hlh-30(tm1978)* | 20.41 ±0.37 | 24 | -54.69% | 141/150 | <0.0001 | - |
| Wild-type (WT) | 16.45 ±0.34 | 21 | - | 140/150 | - | Fig. 6c |
| *smg-2(qd101)* | 16.10 ±0.36 | 19 | -2.13% | 125/150 | 0.4923 | Fig. 6c |
| *sqIs17[hlh-30p::hlh-30::GFP; rol-6(su1006)]* | 20.72 ±0.53 | 23 | 25.96% | 118/120 | <0.0001 | Fig. 6c |
| *smg-2(qd101); sqIs17[hlh-30p::hlh-30::GFP; rol-6(su1006)]* | 15.14 ±0.31 | 19 | -7.96% | 134/150 | 0.0021 | Fig. 6c |
| WT | 18.20 ±0.64 | 26 | - | 127/150 | - | - |
| *smg-2(qd101)* | 15.85 ±0.46 | 19 | -12.91% | 122/150 | <0.01 | - |
| *sqIs17[hlh-30p::hlh-30::GFP; rol-6(su1006)]* | 20.74 ±0.59 | 26 | 13.96% | 146/150 | <0.05 | - |
| *smg-2(qd101); sqIs17[hlh-30p::hlh-30::GFP; rol-6(su1006)]* | 16.06 ±0.35 | 19 | -11.76% | 134/150 | <0.001 | - |
| WT | 16.18 ±0.46 | 21 | - | 114/151 | - | Fig. 6d |
| *hlh-30(tm1978)* | 15.70 ±0.35 | 19 | -2.97% | 133/150 | 0.183 | Fig. 6d |
| *yhEx330[smg-1p::smg-1::gfp; odr-1p::RFP]* | 18.48 ±0.43 | 21 | 14.22% | 123/150 | <0.01 | Fig. 6d |
| *hlh-30(tm1978); yhEx330[smg-1p::smg-1::gfp; odr-1p::RFP]* | 16.24 ±0.27 | 19 | 0.37% | 128/150 | 0.3583 | Fig. 6d |
| *daf-2(e1370)* | 45.51  ±0.84 | 51 | - | 103/125 | - | Fig.6e |
| *daf-2(e1370) pfd-6* RNAi | 27.17  ±0.77 | 32 | -40.30% | 76/114 | <0.0001 | Fig.6e |
| *daf-2(e1370); hlh-30(tm1978)* | 31.26  ±0.61 | 36 | -31.31% | 97/100 | <0.0001 | Fig.6e |
| *daf-2(e1370); hlh-30(tm1978) pfd-6* RNAi | 27.21  ±0.64 | 32 | -40.21% | 107/125 | <0.0001 | Fig.6e |
| *daf-2(e1370)* | 45.69  ±1.21 | 51 | - | 53/75 | - |  |
| *daf-2(e1370) pfd-6* RNAi | 25.32  ±0.62 | 31 | -44.58% | 93/125 | <0.0001 |  |
| *daf-2(e1370); hlh-30(tm1978)* | 30.23  ±0.41 | 33 | -33.84% | 119/125 | <0.0001 |  |
| *daf-2(e1370); hlh-30(tm1978) pfd-6* RNAi | 24.53  ±0.56 | 28 | -46.31% | 110/125 | <0.0001 |  |
| WT | 18.22±0.38 | 21 | - | 117/125 | - | Fig.6f |
| *pfd-6* RNAi | 15.99±0.28 | 19 | -12.24% | 103/125 | <0.0001 | Fig.6f |
| *sqIs17[hlh-30p::hlh-30::GFP; rol-6(su1006)]* | 20.69±0.48 | 24 | 13.56% | 122/125 | <0.0001 | Fig.6f |
| *sqIs17[hlh-30p::hlh-30::GFP; rol-6(su1006)] pfd-6* RNAi | 15.96±0.29 | 17 | -12.40% | 108/125 | <0.0001 | Fig.6f |
| WT | 20.29±0.57 | 24 | - | 76/125 | - |  |
| *pfd-6* RNAi | 16.25±0.49 | 18 | -19.91% | 54/100 | <0.0001 |  |
| *sqIs17[hlh-30p::hlh-30::GFP; rol-6(su1006)]* | 20.77±0.5 | 21 | 2.37% | 92/125 | 0.6704 |  |
| *sqIs17[hlh-30p::hlh-30::GFP; rol-6(su1006)] pfd-6* RNAi | 14.94±0.5 | 15 | -26.37% | 76/125 | <0.0001 |  |

Different experimental sets are distinguished by double-solid lines, and biological replicates within the same experimental set are distinguished by single-solid lines. Biological replicate experiments were performed in parallel if not stated otherwise, and statistical analysis was performed within each replicate experiment. Percent lifespan changes and *p* values were calculated against *daf-2(e1370)* or WT conditions. *p* values were calculated using Mantel-Cox log-rank test.
